# Trajectory following and stabilization control of fully actuated AUV using inverse kinematics and self-tuning fuzzy PID

**DOI:** 10.1371/journal.pone.0179611

**Published:** 2017-07-06

**Authors:** Mohanad M. Hammad, Ahmed K. Elshenawy, M.I. El Singaby

**Affiliations:** Electrical and Control Engineering, Arab Academy for Science, Technology and Maritime Transport, Alexandria, Egypt; Chongqing University, CHINA

## Abstract

In this work a design for self-tuning non-linear Fuzzy Proportional Integral Derivative (FPID) controller is presented to control position and speed of Multiple Input Multiple Output (MIMO) fully-actuated Autonomous Underwater Vehicles (AUV) to follow desired trajectories. Non-linearity that results from the hydrodynamics and the coupled AUV dynamics makes the design of a stable controller a very difficult task. In this study, the control scheme in a simulation environment is validated using dynamic and kinematic equations for the AUV model and hydrodynamic damping equations. An AUV configuration with eight thrusters and an inverse kinematic model from a previous work is utilized in the simulation. In the proposed controller, Mamdani fuzzy rules are used to tune the parameters of the PID. Nonlinear fuzzy Gaussian membership functions are selected to give better performance and response in the non-linear system. A control architecture with two feedback loops is designed such that the inner loop is for velocity control and outer loop is for position control. Several test scenarios are executed to validate the controller performance including different complex trajectories with and without injection of ocean current disturbances. A comparison between the proposed FPID controller and the conventional PID controller is studied and shows that the FPID controller has a faster response to the reference signal and more stable behavior in a disturbed non-linear environment.

## Introduction

AUV is considered one of the most challenging and difficult fields of research. Contemporary markets are looking to improve this field of research because of its great commercial importance and need [1]. Recently, AUV usage expanded to include seabed mapping, oceanographic and underwater living species exploration and research [2–4], oil industry pipeline inspection and maintenance [5–7], search and rescue missions [8], and operations in polluted and shallow water [9, 10].

Controlling an AUV is challenging because of uncertainties in AUV parameters and coefficients, coupled AUV dynamics, and non-linearity of underwater environments due to ocean current disturbances, hydrodynamics drag forces, and uncertain coefficients [11, 12]. Because of this, the conventional linear controllers like PID will only give the desired behavior around certain inputs and disturbances that it is tuned for. That is why the utilization of artificial intelligence to do self-tuning of the PID parameters is required, and will allow the AUV to have a robust control system in the disturbed non-linear environment [13–17].

The desired controller should be robust and adapt to the changes in AUV and environment parameters. It should also be adaptive to the changes in the control performance because of the ocean current disturbance and the variation in AUV dynamics. Thus many control techniques have been proposed for the control of the AUV such as linear controllers [18, 19], Sliding-Mode Controllers (SMC) [20, 21], Fuzzy Logic Control (FLC) [22–26], adaptive Control [27, 28], and neural network-based control [29, 30].

The disadvantages of linear controllers like PID, Linear Quadratic Regulator (LQR) and Linear Quadratic Gaussian (LQG) are that they only have stable performance around specific operating points and are not stable for variations in the environment and AUV parameters. The SMC is considered as an efficient and robust control for high-order complex non-linear systems. The major advantage of sliding mode is its low sensitivity to plant parameter variations and disturbances that do not require exact modeling. However, the implementation of SMC may lead to an undesirable phenomenon of oscillations with finite frequency and amplitude called “chattering” that results in low control accuracy, high wearing of moving mechanical parts, and wasting energy in actuators. The main reason for the chattering is the ignoring of dynamics from actuators and sensors in the system modeling. Some researchers presented approaches for chattering mitigation and suppression [31]. The inverse neural network-based controller disadvantage is that it requires a long time for training, and it is very difficult to obtain a fit generic model as the model can be over-fitted.

Fuzzy logic is a way to make machines more intelligent by enabling them to reason in a fuzzy manner like humans. It was first proposed by Lotfy Zadeh in 1965 [32]. It can deal with uncertain and qualitative decision-making problems. Controllers that combine intelligent and conventional techniques are commonly used in the control of complex dynamic systems. In the design of the traditional controllers like the PID, the knowledge of the system’s realistic physical model is required but are mostly unavailable because of their complexity. Fuzzy controllers are rule-based controllers that benefit from the expertise of human knowledge. They use a reasoning rule base for estimating the required control signal regardless of the system’s physical model knowledge [22, 23]. The main disadvantage of the FLC is that it has a lot of parameters to tune like the ranges and shapes of the membership functions, as opposed to the PID that has only three parameters. Beside it requires much more computation time than the conventional PID because of the complex operations. The FLC doesn’t have much better characteristics in time domain than the PID but the main advantage is that it can work with non-linear systems [33].

In this study a combination of the FLC and PID is utilized to obtain an enhanced control response with the PID controller under the supervision of the FLC system. This method combines the simple mathematical equations and low computation time of the PID controller with the ability of the FLC to tune and adapt the PID parameters so that they may work with non-linear systems. A comparison with the conventional PID is done to demonstrate the time-domain performance and the function of the self-tuned Fuzzy PID controller (FPID). In the design of the fuzzy membership functions, a combination of trapezoidal, triangular inputs and Gaussian functions for outputs are utilized. In Khodayari’s 2015 study [34] a similar approach has been introduced, but the inputs have only triangular membership functions and the fuzzy tuning is used for non-fully actuated AUV. In this work the fuzzy rules differ from the study in [34] as they provide a better tuning behaviour for the fully actuated system in the disturbed non-linear environment. The aim of using Gaussian membership functions is to obtain a non-linear response because of the non-linearity of the AUV system dynamics and hydrodynamics [35]. The AUV configuration along with the inverse kinematics and control architecture used in this research is demonstrated and presented in our previous work [18]. The advantage of this configuration is that we are able to have a fully-actuated AUV in which all degrees of freedom are controllable. This is achieved by accurately controlling the angular speed of each thruster independently based on the reference trajectory signals. This feature makes the AUV capable of tasks that require precise stabilization to ocean current disturbances like underwater pipeline maintenance and path-following in very narrow spaces, such as in the exploration of drowned ships.

This paper is organized as follows. Section “Kinematics and Coordinate Systems” explains the coordinate systems and kinematic modeling. Section “Configuration and Inverse Kinematics control model” shows the AUV configuration, thruster model, and thrusters inverse kinematic equations. In Section “Dynamic Model”, the AUV and environment dynamic equations are discussed. In Section “Control Design”, the proposed control architecture and design is demonstrated. In Section “Results”, a comparative simulation’s results and analysis of the robustness of the controllers are presented.

## Kinematics and coordinate systems

**Fig 1:**
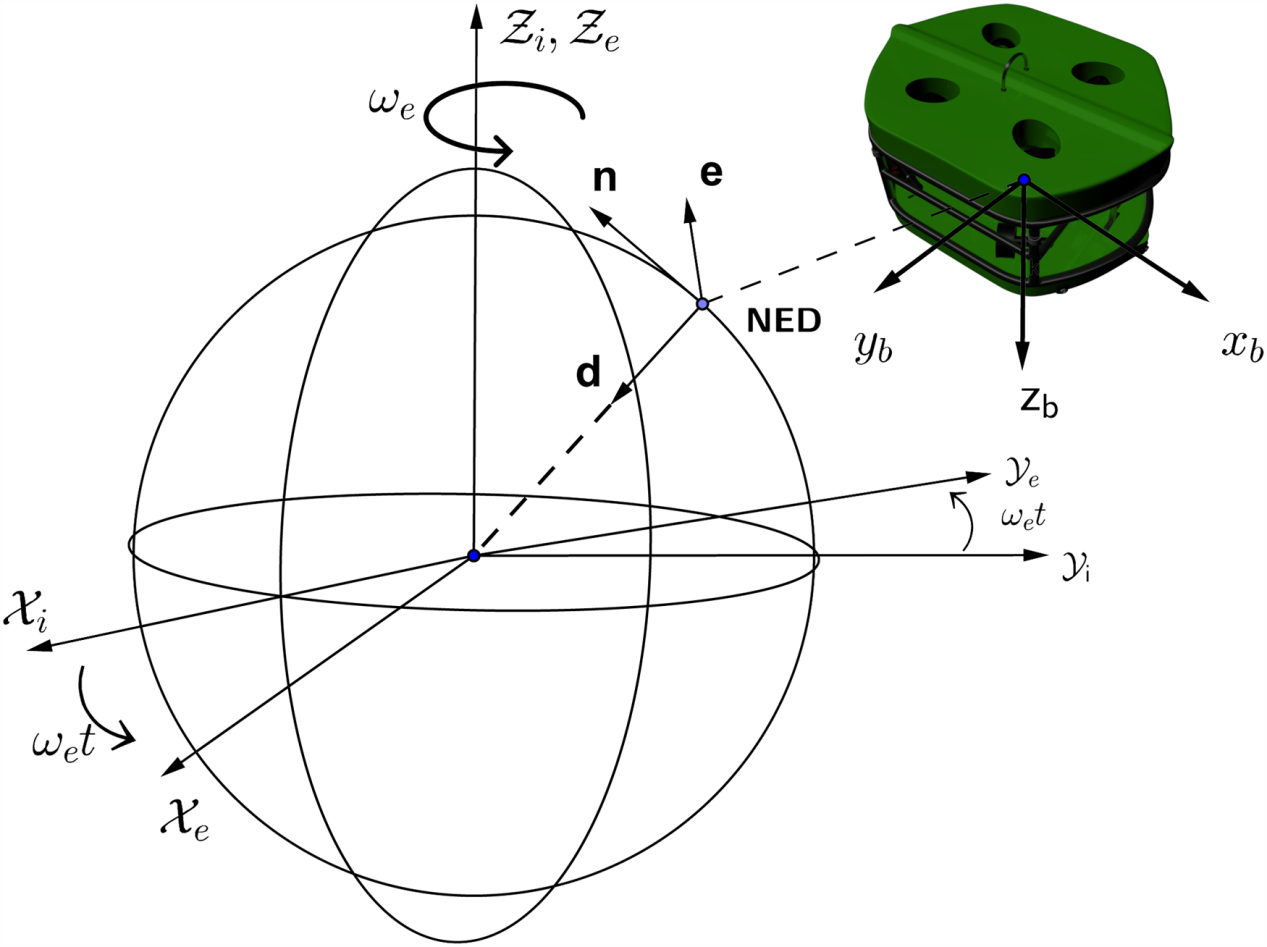
Coordinate systems.

The frames of references and coordinate systems used in marine navigation systems are explained in Fossen’s 2002 study [36] and also shown in Fig 1, the Earth-Centered Inertial frame (ECI/ i-frame) represented by [*x*_*i*_, *y*_*i*_, *z*_*i*_], the Earth-centered Earth-Fixed reference frame (ECEF/ e-frame) represented by [*x*_*e*_, *y*_*e*_, *z*_*e*_], the North-East-Down coordinate system (NED/ n-frame) represented by [*n*, *e*, *d*], and finally the body-fixed reference frame (b-frame) represented by [*x*_*b*_, *y*_*b*_, *z*_*b*_].

**Fig 2:**
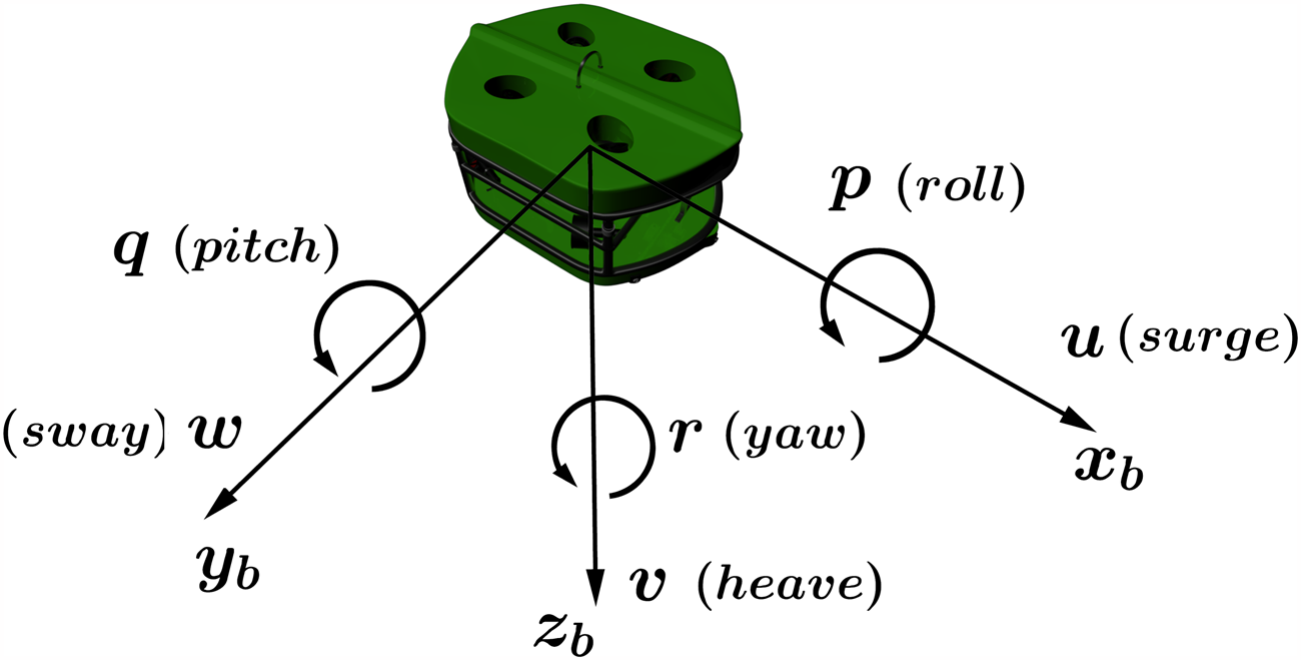
Motion variables for a marine vessel (SNAME 1950).

The ECI frame is a non-accelerating frame in which its origin is at the center of the Earth. The ECEF frame also has its origin fixed at the center of the Earth, but its axes is rotating relative to the ECI frame, which is fixed in space. The ECEF frame rotates at angular rate *ω*_*e*_ = 7.2921.10^−5^ rad/sec [37]. The NED frame is defined as the tangent plane on the surface of the Earth. For this system, the x-axis points north, the y-axis points east, and the z-axis points to the center of the Earth. The location of the n-frame relative to the e-frame is determined by using angles *l* and *μ* to denote longitude and latitude, respectively. The body-fixed frame is a moving frame with the AUV and the origin “O” of the b-frame is usually coinciding with the center of gravity (CG). For marine vessels, the body axes [*x*_*b*_, *y*_*b*_, *z*_*b*_] are chosen to coincide with the principal axes of inertia as shown in Fig 2.

The motion variables for a marine vessel underwater are represented using six parameters such that the first three parameters describe the translational motions in x, y, and z coordinates while the last three describe the rotations and orientations around the x, y, and z axes as shown in Fig 2 and SNAM’s 1952 study [38]. Table 1 defines each variable.

**Table 1:** The notation of SNAME (1950) for marine vessels.

DOF		Forces and Moments (b-frame)	Velocities and Angular Rates (b-frame)	Position and Euler Angles (n-frame)
1	motions in the x-direction (surge)	X	u	*x*
2	motions in the y-direction (sway)	Y	v	*y*
3	motions in the z-direction (heave)	Z	w	*z*
4	rotation about the x-axis (roll, heel)	K	p	*ϕ*
5	rotation about the y-axis (pitch, trim)	M	q	*θ*
6	rotation about the z-axis (yaw)	N	r	*ψ*

In this work, we assumed that the ECEF frame is fixed and the NED frame will be the inertial frame tangent to the surface of the Earth. This is because the angular speed of the Earth is very small, and the AUV application studied in this paper is low-speed and works within short distances. As a result, the Coriolis effect is negligible. The position and orientation of the AUV will be described in the NED inertial coordinates, while the linear velocities, angular velocities, forces, and moments will be described in the body-fixed frame coordinates as shown in Eqs (1), (2) and (3).

$$\begin{array}{c} P^{n}=[\begin{array}{c} n\\ e\\ d \end{array}]\in R^{3},\Theta =[\begin{array}{c} \phi \\ \theta \\ \psi \end{array}]\in S^{3} \end{array}$$ (1)

$$\begin{array}{c} v_{o}^{b}=[\begin{array}{c} u\\ v\\ w \end{array}]\in R^{3},\omega_{nb}^{b}=[\begin{array}{c} p\\ q\\ r \end{array}]\in R^{3} \end{array}$$ (2)

$$\begin{array}{c} f_{o}^{b}=[\begin{array}{c} X\\ Y\\ Z \end{array}]\in R^{3},m_{o}^{b}=[\begin{array}{c} K\\ M\\ N \end{array}]\in R^{3} \end{array}$$ (3)

Where *P*^*n*^ is the AUV position vector in the NED frame, Θ is Euler angles vector, $v_{o}^{b}$ is body-fixed linear velocity vector, $\omega_{nb}^{b}$ is body-fixed angular velocity vector, $f_{o}^{b}$ is body-fixed forces vector, $m_{o}^{b}$ is body-fixed moments vector. The generalized 6-DOF kinematic equation is as shown in Eq (4), that transforms velocities from body-fixed frame coordinates to NED inertial frame coordinates, such that the transformation matrix in Eq (5) transforms from AUV linear velocities $v_{o}^{b}$ to AUV inertial rates ${\dot{P}}^{n}$, the matrix in Eq (6) transforms from AUV angular velocities $\omega_{nb}^{b}$ to Euler rates $\dot{\Theta }$. The Euler rotation has a disadvantage such that a singularity might happen in the calculations which is called Gimbal Lock phenomenon. This singularity is later solved using quaternions in doing rotations instead of Euler.

$$\begin{array}{c} \left[\begin{array}{c} {\dot{P}}^{n}\\ \dot{\Theta } \end{array}]=[\begin{array}{cc} R_{b}^{n}\left(\Theta \right) & 0_{3x3}\\ 0_{3x3} & T_{\Theta }\left(\Theta \right) \end{array}]\;[\begin{array}{c} v_{o}^{b}\\ \omega_{nb}^{b} \end{array}\right] \end{array}$$ (4)

where;

$$\begin{array}{c} R_{b}^{n}\left(\Theta \right)=[\begin{array}{ccc} c\psi c\theta & -s\psi c\phi +c\psi s\theta & s\psi s\phi +c\psi s\theta c\phi \\ s\psi c\theta & c\psi c\phi +s\psi s\theta s\phi & -c\psi s\phi +s\psi s\theta c\phi \\ -s\theta & c\theta s\phi & c\theta c\phi \end{array}] \end{array}$$ (5)

$$\begin{array}{c} T_{\Theta }\left(\Theta \right)=[\begin{array}{ccc} 1 & s\phi t\theta & c\phi t\theta \\ 0 & c\phi & -s\phi \\ 0 & s\phi /c\theta & c\phi /c\theta \end{array}] \end{array}$$ (6)

## Configuration and inverse kinematics control model

**Fig 3:**
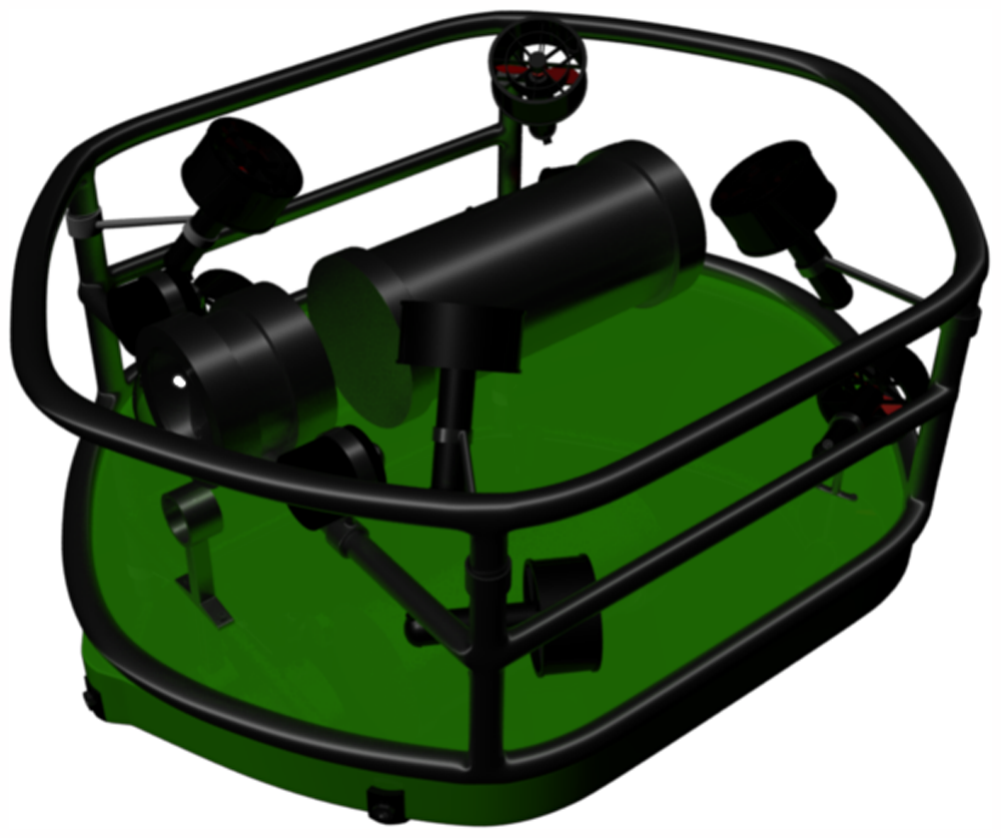
AUV body configuration.

The AUV dimensions are 30cm, 30cm and 40cm for height, width, and length respectively. A total of eight thrusters are used. The thrusters have been mounted in a vectored orientation configuration, and they will be classified into two groups, vertical and horizontal thrusters. The proposed modular thrusters configuration allow the AUV to have a holonomic motion. Such modular configuration approaches have been studied in wheeled mobile robots [39] and has shown more controllability. The configuration is shown in Fig 3. The top and bottom floaters are adjusted such that the AUV is neutrally buoyant underwater.

**Fig 4:**
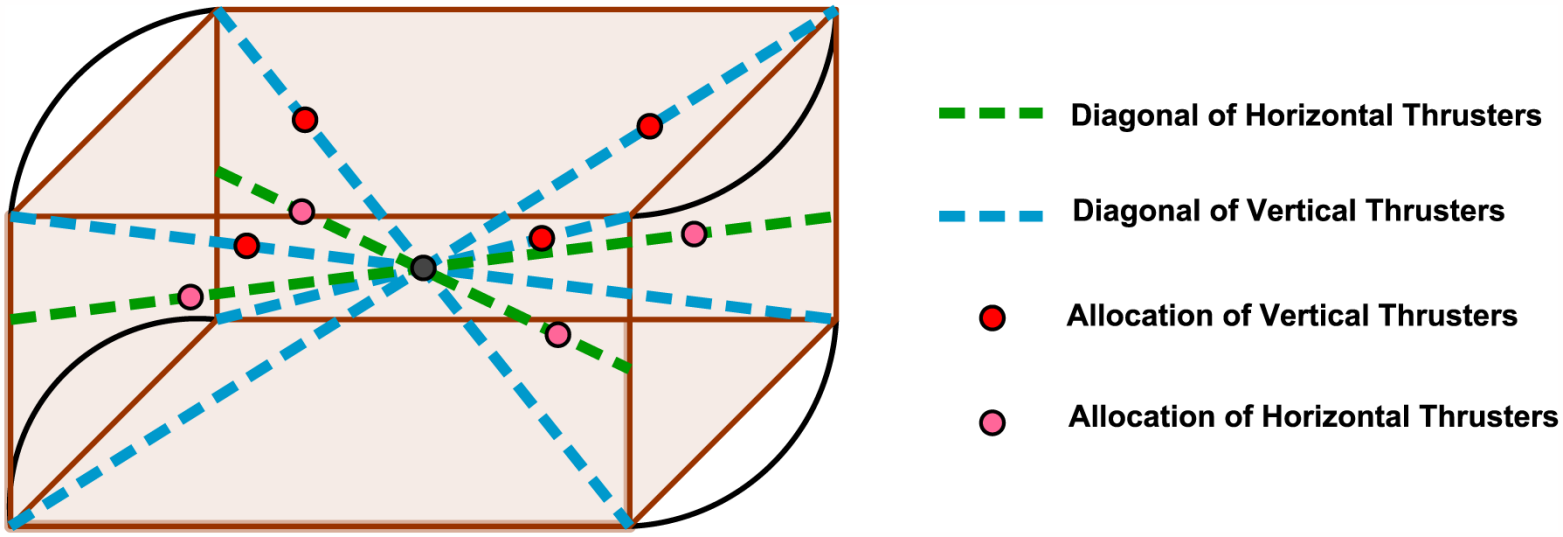
Vessel center point and thrusters allocation.

The aim of the oriented vertical and horizontal thrusters is to obtain a unified center of rotation for the AUV vessel as shown in Fig 4.

**Fig 5:**
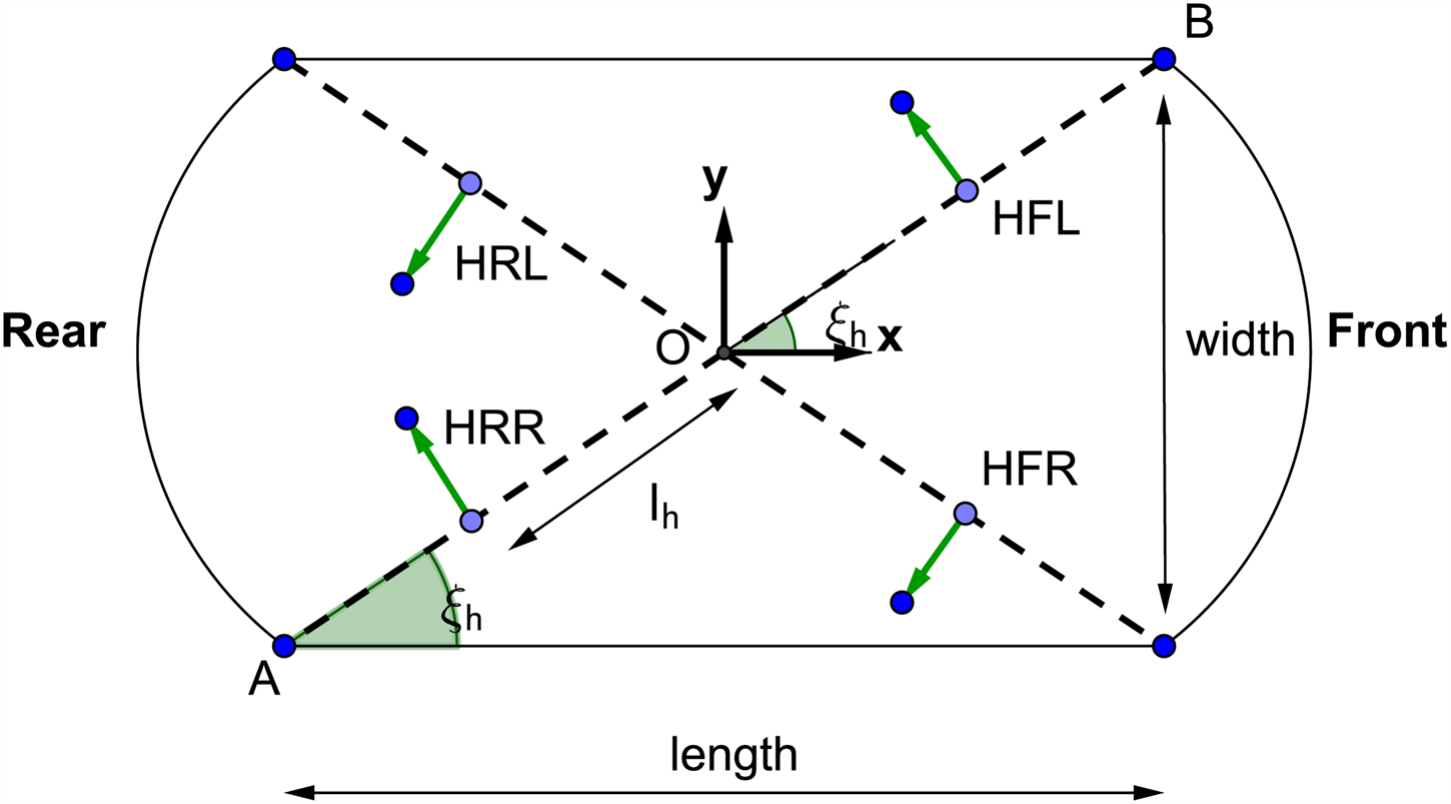
Horizontal thrusters geometrical configuration.

**Fig 6:**
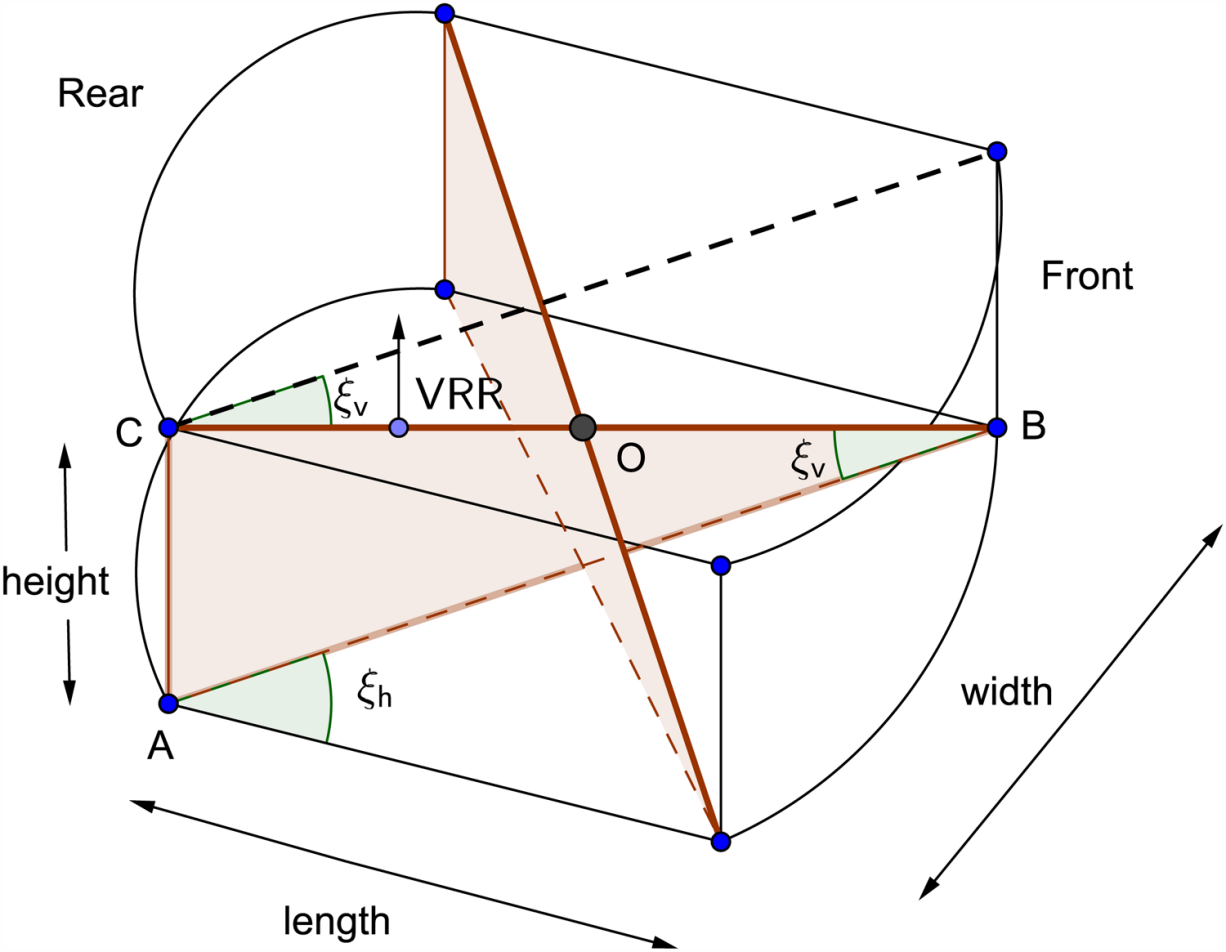
Vertical thrusters geometrical configuration 1.

**Fig 7:**
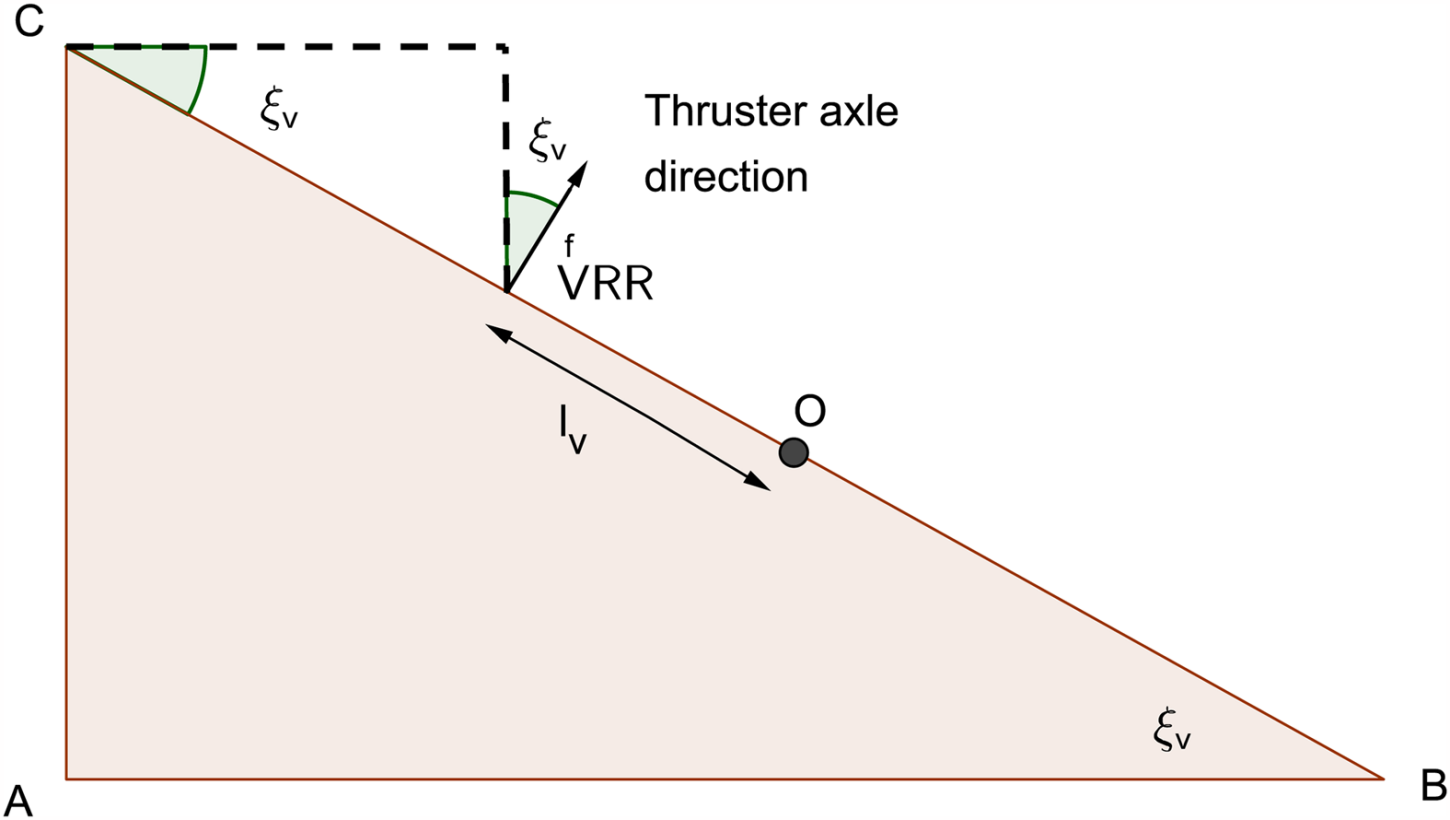
Vertical thrusters geometrical configuration 2.

An inverse kinematic equations that relate resultant AUV vessel velocities in the body-axis frame to the required thruster angular velocities have been developed. With the aid of these equations it was possible to convert the AUV to be fully-actuated. The parameters used in the inverse kinematic model of the horizontal thrusters are demonstrated in Fig 5 where *l_h_* is the distance between the center of the horizontal thruster and the center *O* of the AUV chassis. Figs 6 and 7 show the geometrical configurations of the vertical thrusters where *l_v_* is the distance between the center of the vertical thruster and the center *O* of the AUV chassis. The thrusters are mounted such that the axle is normal to the diagonal where they are located.

The angles *ξ*_*h*_ and *ξ*_*v*_ is calculated using the equations:

$$\begin{array}{c} \xi_{h}=arctan\left(\frac{width}{length}\right) \end{array}$$ (7)

$$\begin{array}{c} \xi_{v}=arctan\left(\frac{height}{\sqrt{width^{2}+length^{2}}}\right) \end{array}$$ (8)

The inverse kinematic control model for the horizontal thrusters are represented by:

$$\begin{array}{c} \left[\begin{array}{c} \omega_{HFL}\\ \omega_{HFR}\\ \omega_{HRR}\\ \omega_{HRL} \end{array}]=\frac{\lambda }{P_{prop}}\;[\begin{array}{ccc} -sec(\xi_{h}) & csc(\xi_{h}) & l_{h}\\ -sec(\xi_{h}) & -csc(\xi_{h}) & -l_{h}\\ -sec(\xi_{h}) & csc(\xi_{h}) & -l_{h}\\ -sec(\xi_{h}) & -csc(\xi_{h}) & l_{h} \end{array}]\;[\begin{array}{c} u\\ v\\ r \end{array}\right] \end{array}$$ (9)

While for the vertical thrusters are represented by:

$$\begin{array}{c} \left[\begin{array}{c} \omega_{VFL}\\ \omega_{VFR}\\ \omega_{VRR}\\ \omega_{VRL} \end{array}]=\frac{\lambda }{P_{prop}}\;[\begin{array}{ccc} sec(\xi_{v}) & l_{v} & -l_{v}\\ sec(\xi_{v}) & -l_{v} & -l_{v}\\ sec(\xi_{v}) & -l_{v} & l_{v}\\ sec(\xi_{v}) & l_{v} & l_{v} \end{array}]\;[\begin{array}{c} w\\ p\\ q \end{array}\right] \end{array}$$ (10)

Where propeller pitch *P*_*prop*_ is the axial distance covered in one revolution of the propeller (m/rev). λ > 0 is a unit-less control factor that is tuned to improve the controller response. The thruster angular velocity is *ω*_*XYZ*_, such that “*X*” stands for Horizontal (H) or Vertical (V), “*Y*” stands for Front (F) or Rear (R), and “*Z*” stands for Left (L) or Right (R).

The rule of thumb to formulate the inverse kinematic control model is to define the role of each thruster in actuating a DOF such that, for example, in case of the Horizontal Front Right (HFR) thruster, The thruster axial motion may lead to surge and sway motions in the AUV besides yaw rotation. It does not contribute to the heave motion since the thruster is normal to the z-axis of the AUV body-frame and can not provide roll and pitch rotations. In the case of a vertical thruster for example the Vertical Rear Left (VRL) thruster, this thruster axial motion may lead to motions in surge, sway, and heave motions besides roll, pitch, and yaw rotations since it has polar and azimuthal angles in x-y-z planes. However, in generating the control signals, we don’t need to command vertical thrusters to do surge and sway motions and yaw rotation, because it will be accompanied with undesired rotations and hence unstable movements. Vertical thrusters are commanded only if a heave motion or roll and pitch rotations are required. The controller will correct the undesired surge and sway movements using equations for horizontal thrusters and the feedback loop.

## Dynamic model

### Rigid body dynamics

The rigid body dynamic equations used in this study are derived from and formulated by [36] using Newton-Euler method.

The final general equations for translational motions are:

$$\begin{array}{c} X=m[\dot{u}-vr+wq-x_{g}\left(q^{2}+r^{2}\right)+y_{g}\left(pq-\dot{r}\right)+z_{g}\left(pr+\dot{q}\right)] \end{array}$$ (11)

$$\begin{array}{c} Y=m[\dot{v}-wp+ur-y_{g}\left(r^{2}+p^{2}\right)+z_{g}\left(qr-\dot{p}\right)+x_{g}\left(qp+\dot{r}\right)] \end{array}$$ (12)

$$\begin{array}{c} Z=m[\dot{w}-uq+vp-z_{g}\left(p^{2}+q^{2}\right)+x_{g}\left(rp-\dot{q}\right)+y_{g}\left(rq+\dot{p}\right)] \end{array}$$ (13)

And for rotational motions are:

$$\begin{array}{c} \begin{array}{ccc} K & = & I_{xx}\dot{p}+\left(I_{zz}-I_{yy}\right)qr-\left(\dot{r}+pq\right)I_{xz}+\left(r^{2}-q^{2}\right)I_{yz}+\left(pr-\dot{q}\right)I_{xy}\\ & + & m[y_{g}\left(\dot{w}-uq+vp\right)-z_{g}\left(\dot{v}-wp+ur\right)] \end{array} \end{array}$$ (14)

$$\begin{array}{c} \begin{array}{ccc} M & = & I_{yy}\dot{q}+\left(I_{xx}-I_{zz}\right)rp-\left(\dot{p}+qr\right)I_{xy}+\left(p^{2}-r^{2}\right)I_{zx}+\left(qp-\dot{r}\right)I_{yz}\\ & + & m[z_{g}\left(\dot{u}-vr+wq\right)-x_{g}\left(\dot{w}-uq+vp\right)] \end{array} \end{array}$$ (15)

$$\begin{array}{c} \begin{array}{ccc} N & = & I_{zz}\dot{r}+\left(I_{yy}-I_{xx}\right)pq-\left(\dot{q}+rp\right)I_{yz}+\left(q^{2}-p^{2}\right)I_{xy}+\left(rq-\dot{p}\right)I_{zx}\\ & + & m[x_{g}\left(\dot{v}-wp+ur\right)-y_{g}\left(\dot{u}-vr+wq\right)] \end{array} \end{array}$$ (16)

Such that *“m”* is the mass of the vessel in kg and the $r_{g}^{b}={\left[x_{g},y_{g},z_{g}\right]}^{T}$ is the vector from the vessel origin *O* to the center of gravity (*CG*) decomposed in b-frame and *I*_*o*_ is the inertia tensor of the vessel which is described by:

$$\begin{array}{c} I_{o}=[\begin{array}{ccc} I_{xx} & -I_{xy} & -I_{xz}\\ -I_{yx} & I_{yy} & -I_{yz}\\ -I_{zx} & -I_{zy} & I_{zz} \end{array}] \end{array}$$ (17)

Where *I*_*xx*_, *I*_*yy*_, and *I*_*zz*_ are the moments of inertia about *x*_*b*_, *y*_*b*_, and *z*_*b*_-axes:

$$\begin{array}{c} \begin{array}{ccc} I_{xx} & = & \int_{V}\left(y^{2}+z^{2}\right)\rho_{m}dV\\ I_{yy} & = & \int_{V}\left(x^{2}+z^{2}\right)\rho_{m}dV\\ I_{zz} & = & \int_{V}\left(x^{2}+y^{2}\right)\rho_{m}dV \end{array} \end{array}$$ (18)

And *I*_*xy*_ = *I*_*yx*_, *I*_*xz*_ = *I*_*zx*_, and *I*_*zy*_ = *I*_*yz*_ are the products of inertia:

$$\begin{array}{c} \begin{array}{ccc} I_{xy} & = & \int_{V}xy\;\rho_{m}dV\\ I_{yz} & = & \int_{V}yz\;\rho_{m}dV\\ I_{xz} & = & \int_{V}xz\;\rho_{m}dV \end{array} \end{array}$$ (19)

Further simplification for the 6-DOF rigid body equations of motion is done such that it is assumed that the vessel is already buoyant and the center of buoyancy and body-axis frame [*O*, *x*_*b*_, *y*_*b*_, *z*_*b*_] coincides with the CG and principal axis of inertia, hence $r_{g}^{b}={\left[0,0,0\right]}^{T}$ and *I*_*o*_ = *diag*(*I*_*xx*_, *I*_*yy*_, *I*_*zz*_). As a result, the simplified equations of motion is defined as:

$$\begin{array}{c} \begin{array}{ccc} X & = & m[\dot{u}-vr+wq]\\ Y & = & m[\dot{v}-wp+ur]\\ Z & = & m[\dot{w}-uq+vp]\\ K & = & I_{xx}\dot{p}+\left(I_{zz}-I_{yy}\right)qr\\ M & = & I_{yy}\dot{q}+\left(I_{xx}-I_{zz}\right)rp\\ N & = & I_{zz}\dot{r}+\left(I_{yy}-I_{xx}\right)pq \end{array} \end{array}$$ (20)

According to [40] the rigid body dynamics can be expressed in vectorial form as:

$$\begin{array}{c} M_{RB}\dot{\nu }+C_{RB}\left(\nu \right)\nu =\tau_{RB} \end{array}$$ (21)

Where *ν* = [*u*, *v*, *w*, *p*, *q*, *r*]^*T*^ is the body-fixed linear and angular velocity vector. *τ*_*RB*_ = [*X*, *Y*, *Z*, *K*, *M*, *N*]^*T*^ is a generalized vector of external forces and moments. *M*_*RB*_ and *C*_*RB*_ will be referred to as the rigid body inertia, and Coriolis and centrifugal matrices, respectively.

$$\begin{array}{c} \begin{array}{ccc} M_{RB} & = & \left[\begin{array}{cc} mI_{3\times 3} & -mS(r_{g}^{b})\\ mS(r_{g}^{b}) & I_{o} \end{array}\right]\\ & = & \left[\begin{array}{cccccc} m & 0 & 0 & 0 & mz_{g} & -my_{g}\\ 0 & m & 0 & -mz_{g} & 0 & mx_{g}\\ 0 & 0 & m & my_{g} & -mx_{g} & 0\\ 0 & -mz_{g} & my_{g} & I_{xx} & -I_{xy} & -I_{xz}\\ mz_{g} & 0 & -mx_{g} & -I_{yx} & I_{yy} & -I_{yz}\\ -my_{g} & mx_{g} & 0 & -I_{zx} & -I_{zy} & I_{zz} \end{array}\right] \end{array} \end{array}$$ (22)

$$\begin{array}{c} M_{RB}=M_{RB}^{T}=[\begin{array}{cc} m_{11} & m_{12}\\ m_{21} & m_{22} \end{array}]<0 \end{array}$$ (23)

*C*_*RB*_ can be calculated from system inertia matrix. Such that:

$$\begin{array}{c} C_{RB}=[\begin{array}{cc} 0_{3\times 3} & -S(m_{11}\nu_{1}+m_{12}\nu_{2})\\ -S(m_{11}\nu_{1}+m_{12}\nu_{2}) & -S(m_{21}\nu_{1}+m_{22}\nu_{2}) \end{array}] \end{array}$$ (24)

Where *ν*_1_ = [*u*, *v*, *w*] and *ν*_2_ = [*p*, *q*, *r*]. And expression *S*(.) denotes a skew-symmetric matrix or the cross operator such that:

$$\begin{array}{c} \begin{array}{ccc} \vec{a}\times & = & S(\vec{a})\\ & = & \left[\begin{array}{ccc} 0 & -a_{3} & a_{2}\\ a_{3} & 0 & -a_{1}\\ -a_{2} & a_{1} & 0 \end{array}\right] \end{array} \end{array}$$ (25)

### Hydrodynamics

The hydrodynamic damping forces affecting underwater vehicles dynamics contain both drag and lift forces. However, the AUV works at low speeds so the lift force could be neglected because it has effect only at high speeds. As a result, only the drag forces will be considered. D’Alambert’s paradox states that that no hydrodynamic forces act on a solid moving completely submerged with constant velocity in a non-viscous fluid. But in a viscous fluid, frictional forces are present such that the system is not conservative with respect to energy. The drag forces can be separated into linear and non-linear terms, *D*(*ν*) = *D*_*l*_ + *D*_*n*_(*ν*), where *D*_*l*_ is linear drag forces and *D*_*n*_(*ν*) is non-linear drag forces. Since it is assumed that the vehicle body has symmetry about all planes, then *D*_*l*_ can be expressed as:

$$\begin{array}{c} D_{l}=[\begin{array}{cccccc} X_{u} & 0 & 0 & 0 & 0 & 0\\ 0 & Y_{v} & 0 & 0 & 0 & 0\\ 0 & 0 & Z_{w} & 0 & 0 & 0\\ 0 & 0 & 0 & K_{p} & 0 & 0\\ 0 & 0 & 0 & 0 & M_{q} & 0\\ 0 & 0 & 0 & 0 & 0 & N_{r} \end{array}] \end{array}$$ (26)

$$\begin{array}{c} \begin{array}{ccc} X_{l} & = & X_{u}\;u\\ Y_{l} & = & Y_{v}\;v\\ Z_{l} & = & Z_{w}\;w\\ K_{l} & = & K_{p}\;p\\ M_{l} & = & M_{q}\;q\\ N_{l} & = & N_{r}\;r \end{array} \end{array}$$ (27)

Where;

$$\begin{array}{c} \begin{array}{ccc} X_{u} & = & -\frac{1}{2}\rho C_{du}A_{du}\\ Y_{v} & = & -\frac{1}{2}\rho C_{dv}A_{dv}\\ Z_{w} & = & -\frac{1}{2}\rho C_{dw}A_{dw}\\ K_{p} & = & -\frac{1}{16}\rho C_{dp}\;x\;z^{4}\\ M_{q} & = & -\frac{1}{16}\rho C_{dq}\;y\;x^{4}\\ N_{r} & = & -\frac{1}{16}\rho C_{dr}\;z\;x^{4}\; \end{array} \end{array}$$ (28)

Such that *ρ* is the water medium density in *kg*/*m*^3^. *C*_*d*_ is the unit-less drag coefficient which depends on Reynolds number. *A*_*d*_ is the drag contact area in *m*^2^, and *x*, *y*, *and z* are the length, width, and height in meters of the AUV, respectively.

The non-linear drag forces due to vortex shedding in the translational motions can be modeled as shown by:

$$\begin{array}{c} \begin{array}{ccc} X_{nl} & = & -(\frac{1}{2}\rho C_{du}A_{du})\;u\left|u\right|\\ & = & X_{u|u|}u\left|u\right|\\ Y_{nl} & = & -(\frac{1}{2}\rho C_{dv}A_{dv})\;v\left|v\right|\\ & = & Y_{v|v|}v\left|v\right|\\ Z_{nl} & = & -(\frac{1}{2}\rho C_{dw}A_{dw})\;w\left|w\right|\\ & = & Z_{w|w|}w\left|w\right| \end{array} \end{array}$$ (29)

The non-linear drag moments due to rotational motions can be modeled as:

$$\begin{array}{c} \begin{array}{ccc} K_{nl} & = & -(\frac{1}{16}\rho C_{dp}\;x\;z^{4})\;p\left|p\right|\\ & = & K_{p|p|}p\left|p\right|\\ M_{nl} & = & -(\frac{1}{16}\rho C_{dq}\;y\;x^{4})\;q\left|q\right|\\ & = & K_{q|q|}q\left|q\right|\\ N_{nl} & = & -(\frac{1}{16}\rho C_{dr}\;z\;x^{4})\;r\left|r\right|\\ & = & K_{r|r|}r\left|r\right| \end{array} \end{array}$$ (30)

The non-linear drag matrix is expressed as:

$$\begin{array}{c} D_{n}\left(\nu \right)=[\begin{array}{cccccc} X_{u|u|}\left|u\right| & 0 & 0 & 0 & 0 & 0\\ 0 & Y_{v|v|}\left|v\right| & 0 & 0 & 0 & 0\\ 0 & 0 & Z_{w|w|}\left|w\right| & 0 & 0 & 0\\ 0 & 0 & 0 & K_{p|p|}\left|p\right| & 0 & 0\\ 0 & 0 & 0 & 0 & M_{q|q|}\left|q\right| & 0\\ 0 & 0 & 0 & 0 & 0 & N_{r|r|}\left|r\right| \end{array}] \end{array}$$ (31)

The vectorial form of the dynamic model including damping forces will be:

$$\begin{array}{c} M_{RB}\dot{\nu }+C_{RB}\left(\nu \right)\nu +D\left(\nu \right)\nu =\tau_{RB} \end{array}$$ (32)

Such that *D*(*ν*) is the damping matrix.

### Gravitational and buoyancy matrix

**Fig 8:**
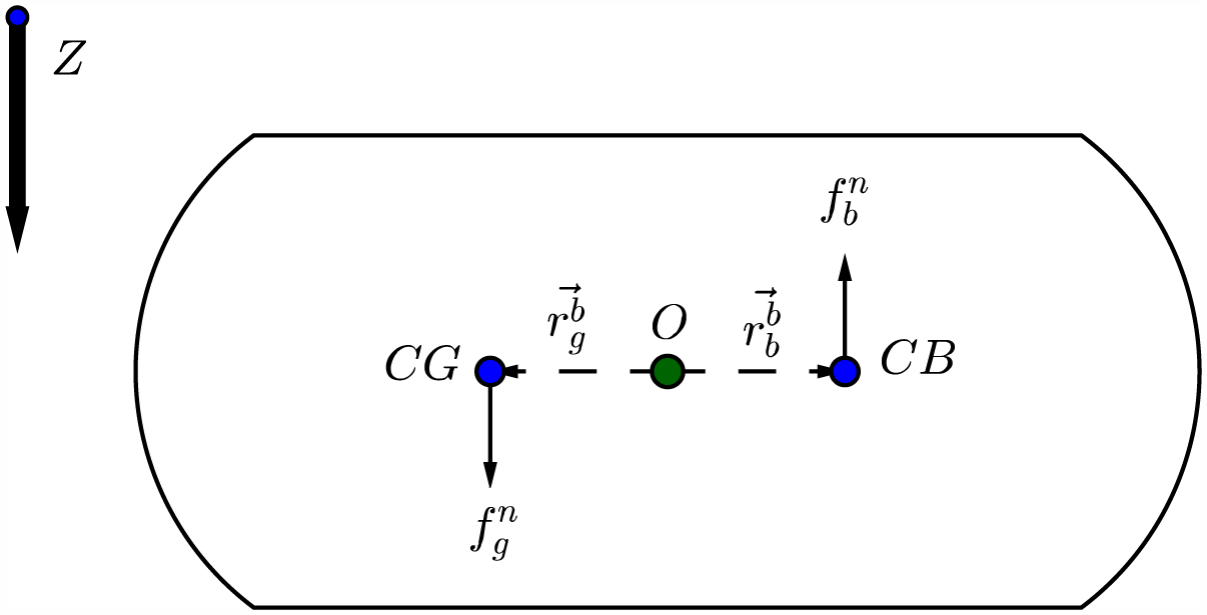
Gravitational and buoyancy forces acting on center of gravity and center of buoyancy of an underwater vessel.

Besides mass and damping forces, the underwater vehicles will also be affected by gravity and buoyancy forces. In hydrodynamics terminology these are called restoring forces as shown in [36]. As shown in Fig 8 the gravitational force $f_{g}^{n}$ will act through the center of gravity (CG) defined by $r_{g}^{b}=\left[x_{g},y_{g},z_{g}\right]$ while the buoyancy force $f_{b}^{n}$ will act through the center of buoyancy (CB) of the vessel defined by $r_{b}^{b}=\left[x_{b},y_{b},z_{b}\right]$.

According to the SNAME (1950) notation [38], the submerged weight of the body and buoyancy force are defined as:

$$\begin{array}{c} W=mg,\;\;\;B=\rho g\nabla \end{array}$$ (33)

Therefore;

$$\begin{array}{c} f_{g}^{n}=[\begin{array}{c} 0\\ 0\\ W \end{array}],\;\;\;f_{b}^{n}=-[\begin{array}{c} 0\\ 0\\ B \end{array}] \end{array}$$ (34)

The weight and buoyancy force can be transformed to the body-fixed coordinate system by:

$$\begin{array}{c} f_{g}^{b}=R_{b}^{n}{\left(\Theta \right)}^{-1}f_{g}^{n},\;\;\;f_{b}^{b}=R_{b}^{n}{\left(\Theta \right)}^{-1}f_{b}^{n} \end{array}$$ (35)

Finally, the gravitational and buoyancy matrix can be expressed as:

$$\begin{array}{c} g\left(\eta \right)=[\begin{array}{c} f_{g}^{b}+f_{b}^{b}\\ r_{g}^{b}\times f_{g}^{b}+r_{b}^{b}\times f_{b}^{b} \end{array}] \end{array}$$ (36)

Which can be expanded to:

$$\begin{array}{c} g\left(\eta \right)=[\begin{array}{c} \left(W-B)sin(\theta \right)\\ -(W-B)cos(\theta )sin(\phi )\\ -(W-B)cos(\theta )cos(\phi )\\ -\left(y_{g}W-y_{b}B\right)cos\left(\theta \right)cos\left(\phi \right)+\left(z_{g}W-z_{b}B\right)cos\left(\theta \right)sin\left(\phi \right)\\ \left(z_{g}W-z_{b}B\right)sin\left(\theta \right)+\left(x_{g}W-x_{b}B\right)cos\left(\theta \right)cos\left(\phi \right)\\ -\left(x_{g}W-x_{b}B\right)cos\left(\theta \right)sin\left(\phi \right)-\left(y_{g}W-y_{b}B\right)sin\left(\theta \right) \end{array}] \end{array}$$ (37)

In this work it is assumed that both centers of gravity and buoyancy are coincided at origin *O* of the vessel $r_{g}^{b}={\left[0,0,0\right]}^{T}$ and $r_{b}^{b}={\left[0,0,0\right]}^{T}$, and the vessel is neutrally buoyant *W* = *B*. Therefore;

$$\begin{array}{c} g\left(\eta \right)=[\begin{array}{c} 0_{6x1} \end{array}] \end{array}$$ (38)

The vectorial form including the gravitation and buoyancy forces will be:

$$\begin{array}{c} M_{RB}\dot{\nu }+C_{RB}\left(\nu \right)\nu +D\left(\nu \right)\nu +g\left(\eta \right)=\tau_{RB} \end{array}$$ (39)

Such that *g*(*η*) is the vector of gravitational/ buoyancy forces and moments.

### Ocean current and disturbances

As explained in [41], ocean currents are horizontal and vertical circulation systems of ocean waters produced by gravity, wind friction, and water density variation in different parts of the ocean. The oceans are conveniently divided into two water spheres, the cold and warm water spheres. Since the Earth is rotating, the Coriolis force will try to turn the major currents to the east in the northern hemisphere and west in the southern hemisphere. Finally, the major ocean circulations will also have a tidal component arising from planetary interactions and gravity. In coastal regions the tidal speeds can reach 2–3 m/s, which is considered a very high speed.

Ocean currents’ forces on marine crafts can be accounted for by replacing the generalized velocity vector in the hydrodynamic terms with relative velocities:

$$\begin{array}{c} \nu_{r}=\nu -\nu_{c} \end{array}$$ (40)

**Fig 9:**
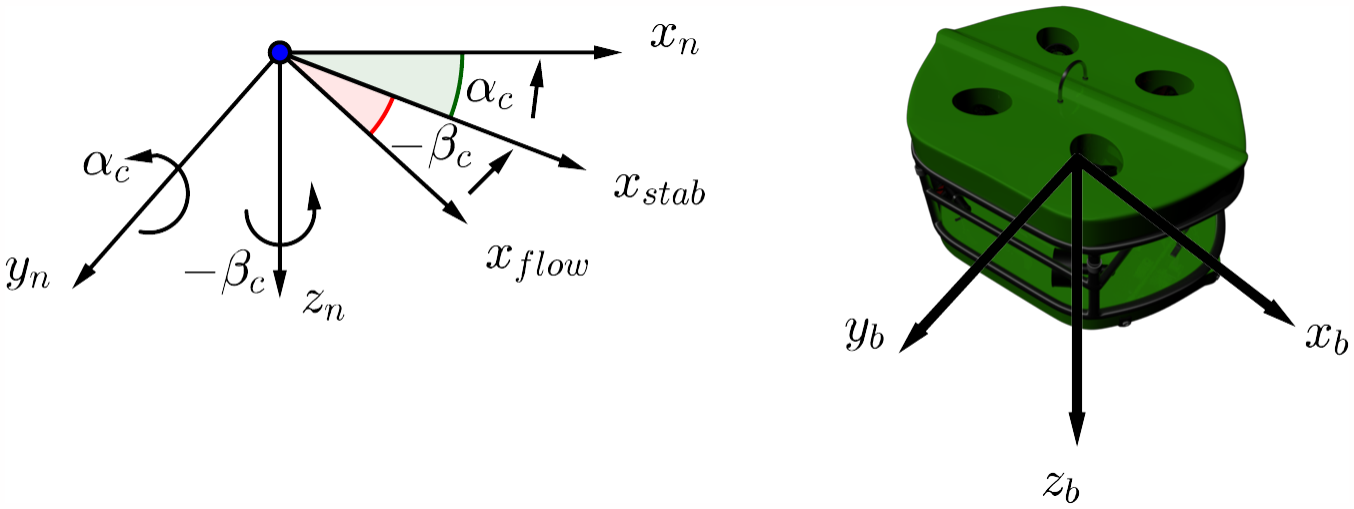
Angle of attack and side-slip angle for a marine craft.

The ocean current speed is denoted by *V*_*c*_ while its direction relative to the moving vessel is expressed by angle of attack *α*_*c*_ and side-slip angle *β*_*c*_ as shown in Fig 9.

Hence the vectorial form dynamic model including ocean current disturbances will be:

$$\begin{array}{c} M_{RB}{\dot{\nu }}_{r}+C_{RB}\left(\nu_{r}\right)\nu_{r}+D\left(\nu_{r}\right)\nu_{r}+g\left(\eta \right)=\tau_{RB} \end{array}$$ (41)

For computer simulations, the ocean current speed and direction can be generated using first order Gauss-Markov processes:

$$\begin{array}{c} {\dot{V}}_{c}+\mu_{1}V_{c}=w_{1} \end{array}$$ (42)

$$\begin{array}{c} {\dot{\alpha }}_{c}+\mu_{2}\alpha_{c}=w_{2} \end{array}$$ (43)

$$\begin{array}{c} {\dot{\beta }}_{c}+\mu_{3}\beta_{c}=w_{3} \end{array}$$ (44)

Where *w*_*i*_ (*i* = 1, 2, 3) are zero-mean Gaussian white noise processes, and *μ*_*i*_ ≥ 0 (*i* = 1, 2, 3) are constants. If *μ*_1_ = *μ*_2_ = *μ*_3_ = 0 then the models are reduced to a random walks corresponding to the time integration of the white noise. A limitation shall be applied to the integration process to limit the current speed:

$$\begin{array}{c} V_{min}\leq V_{c}\left(t\right)\leq V_{max} \end{array}$$ (45)

A 3-D irrotational ocean current model is obtained by transforming the ocean current speed *V*_*c*_ and directions (*α*_*c*_; *β*_*c*_) from current flow axes to NED velocities:

$$\begin{array}{c} \begin{array}{ccc} v_{c}^{n} & = & R_{y,\alpha_{c}}^{T}R_{z,-\beta_{c}}^{T}\;[\begin{array}{c} V_{c}\\ 0\\ 0 \end{array}]\\ & = & \left[\begin{array}{c} V_{c}\;cos\left(\alpha_{c}\right)\;cos\left(\beta_{c}\right)\\ V_{c}\;sin\left(\beta_{c}\right)\\ V_{c}\;sin\left(\alpha_{c}\right)\;cos\left(\beta_{c}\right) \end{array}\right] \end{array} \end{array}$$ (46)

Where;

$$\begin{array}{c} R_{y,\alpha_{c}}=[\begin{array}{ccc} cos(\alpha_{c}) & 0 & sin(\alpha_{c})\\ 0 & 1 & 0\\ -sin(\alpha_{c}) & 0 & cos(\alpha_{c}) \end{array}] \end{array}$$ (47)

$$\begin{array}{c} R_{y,-\beta_{c}}=[\begin{array}{ccc} cos(\beta_{c}) & sin(\beta_{c}) & 0\\ -sin(\beta_{c}) & cos(\beta_{c}) & 0\\ 0 & 0 & 1 \end{array}] \end{array}$$ (48)

The expression in Eq (46) can be transformed from NED to body-axis frame of the vessel using Euler angle rotation matrix $R_{b}^{n}\left(\Theta \right)$ shown in Eq (5):

$$\begin{array}{c} \left[\begin{array}{c} u_{c}\\ v_{c}\\ w_{c} \end{array}]=R_{b}^{n}{\left(\Theta \right)}^{T}\;[\begin{array}{c} V_{c}\;cos\left(\alpha_{c}\right)\;cos\left(\beta_{c}\right)\\ V_{c}\;sin\left(\beta_{c}\right)\\ V_{c}\;sin\left(\alpha_{c}\right)\;cos\left(\beta_{c}\right) \end{array}\right] \end{array}$$ (49)

### Thruster dynamics

**Fig 10:**
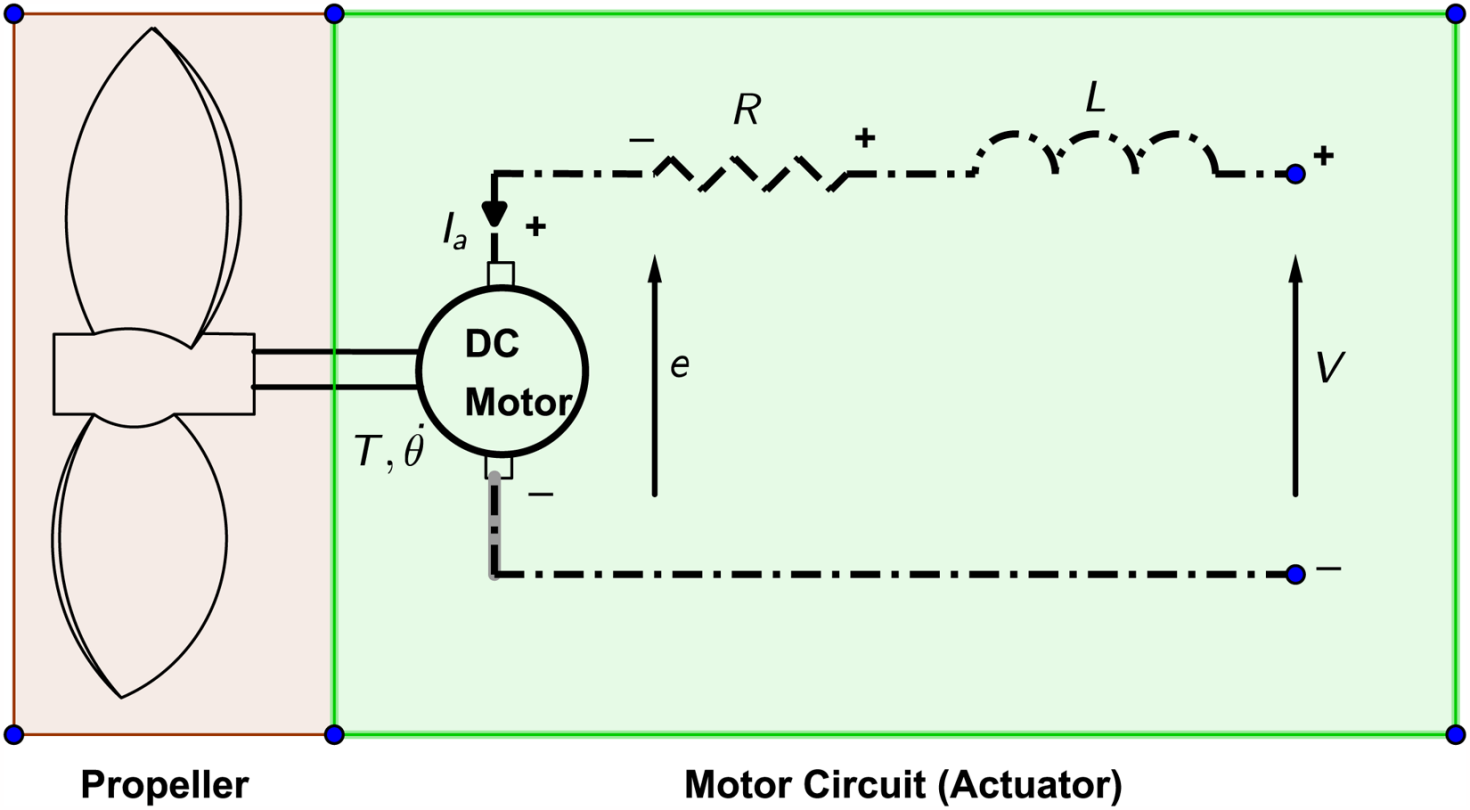
Thruster parts.

The thruster can be separated in to two parts which are the actuator and the propeller as shown in Fig 10.

A model of brushed DC motor is used as an actuator and the dynamic equations are:

$$\begin{array}{c} T=K_{t}i \end{array}$$ (50)

$$\begin{array}{c} e=K_{e}\dot{\theta } \end{array}$$ (51)

$$\begin{array}{c} J\ddot{\theta }+b\dot{\theta }=K_{t}i \end{array}$$ (52)

$$\begin{array}{c} L\frac{di}{dt}+Ri=V-K_{e}\dot{\theta } \end{array}$$ (53)

The abbreviations of the physical parameters are defined in the Table 2.

**Table 2:** DC motor model abbreviations.

Parameter	Abbreviation	Unit
J	moment of inertia of the rotor	*kg*.*m*^2^
b	motor viscous friction constant	N.m.s
*K*_*e*_	electromotive force constant	V/rad/sec
*K*_*t*_	motor torque constant	N.m/Amp
R	electric resistance	Ohm
L	electric inductance	H

The propeller torque and thrust equations as mentioned in [42] are formulated as:

$$\begin{array}{c} Q=K_{Q}\rho D^{5}\left|n\right|n \end{array}$$ (54)

$$\begin{array}{c} T=K_{T}\rho D^{4}\left|n\right|n \end{array}$$ (55)

Where *T* and *Q* are the thrust and torque produced by the propeller, *K*_*T*_ and *K*_*Q*_ are the thrust and torque coefficients, respectively. *ρ* is the water density, *n* is the propeller angular velocity in rev/s. The thrust *T* is the thrust produced in the thruster frame (t-frame).

The resultant forces acting on the AUV body-axis frame in are formulated using force analysis and based on the configuration demonstrated in Figs 5 and 6. They are expressed by:

$$\begin{array}{c} \begin{array}{ccc} X & = & cos\left(\xi_{h}\right)[-T_{HFL}-T_{HFR}-T_{HRR}-T_{HRL}]\\ & + & sin\left(\xi_{v}\right)cos\left(\xi_{h}\right)[-T_{VFL}-T_{VFR}+T_{VRR}+T_{VRL}] \end{array} \end{array}$$ (56)

$$\begin{array}{c} \begin{array}{ccc} Y & = & sin\left(\xi_{h}\right)[T_{HFL}-T_{HFR}+T_{HRR}-T_{HRL}]\\ & + & sin\left(\xi_{v}\right)sin\left(\xi_{h}\right)[-T_{VFL}+T_{VFR}+T_{VRR}-T_{VRL}] \end{array} \end{array}$$ (57)

$$\begin{array}{c} Z=cos\left(\xi_{v}\right)[T_{VFL}+T_{VFR}+T_{VRR}+T_{VRL}] \end{array}$$ (58)

$$\begin{array}{c} K=l_{v}\times [T_{VFL}-T_{VFR}-T_{VRR}+T_{VRL}] \end{array}$$ (59)

$$\begin{array}{c} M=l_{v}\times [-T_{VFL}-T_{VFR}+T_{VRR}+T_{VRL}] \end{array}$$ (60)

$$\begin{array}{c} N=l_{h}\times [T_{HFL}-T_{HFR}-T_{HRR}+T_{HRL}] \end{array}$$ (61)

Where the thrust force is *T*_*XYZ*_, such that *X* stands for Horizontal (H) or Vertical (V), *Y* stands for Front (F) or Rear (R), and *Z* stands for Left (L) or Right (R). *l*_*h*_ is the distance between the center of the horizontal thruster and the center *O* of the AUV chassis. And *l*_*v*_ is the distance between the center of the vertical thruster and the center *O* of the AUV chassis, as shown in the above Figs 6 and 7.

## Control design

**Fig 11:**
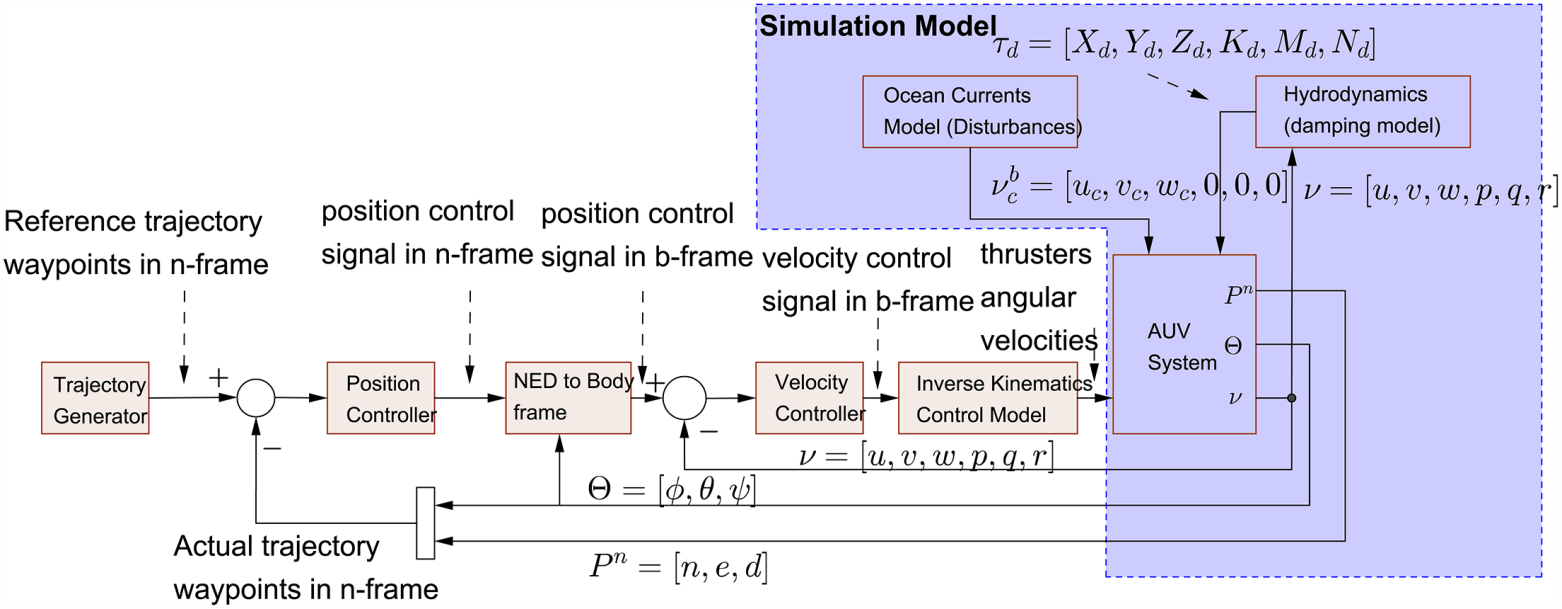
Control system block diagram.

The control system has been design with double control loops. The inner loop is for controlling the AUV b-frame velocity while the outer loop is for controlling the AUV n-frame global position and Euler orientations as shown in Fig 11. The usage of double control loops has two major advantages. First, it provides faster control actions and rapidly attenuating environment disturbances. Second, it gives the control architecture the flexibility to control position and velocity of the AUV independently based on the user need and the required task.

The AUV system, ocean currents model and hydrodynamics model blocks are only used for computer simulation, while the rest of the control architecture should be coded and implemented in the electronic control unit (ECU) to control the real AUV system.

### Trajectory generator

This block is responsible for generating the reference trajectory by which the control system will be tracked. The trajectory is generated by means of way-points at fixed time-steps. Since the underwater system is considered a slow system, a larger fixed time-step will be used such that the AUV has the enough time to reach the desired way-point. Different shapes for trajectories can be used, such as straight line, circle, infinite and Möbius shapes.

For a straight line trajectory in 3D the equation will be:

$$\begin{array}{c} {\vec{k}}_{f}={\vec{k}}_{0}+\left(t\times \vec{h}\right) \end{array}$$ (62)

$$\begin{array}{c} \begin{array}{ccc} x & = & x_{0}+\left(t\times h_{x}\right)\\ y & = & y_{0}+\left(t\times h_{y}\right)\\ z & = & z_{0}+\left(t\times h_{z}\right) \end{array} \end{array}$$ (63)

Where $\vec{h}=\left[h_{x},h_{y},h_{z}\right]$ specifies the direction or the slope of the line, [*x*_0_, *y*_0_, *z*_0_] specifies the initial point of the line, and [*x*, *y*, *z*] specifies the new point of the line.

For a 2D circle shape trajectory the equation will be:

$$\begin{array}{c} \begin{array}{ccc} x & = & R\times cos(\delta )\\ y & = & R\times sin(\delta ) \end{array} \end{array}$$ (64)

Where *R* is the radius and *δ* is the angle in radians. *δ* is incremented by a fixed value at each fixed time-step.

For a 3D Möbius shape trajectory the equation will be:

$$\begin{array}{c} \begin{array}{ccc} x & = & \left(1+\frac{R}{2}cos\left(\frac{\delta }{2}\right)\right)cos\left(\delta \right)\\ y & = & \left(1+\frac{R}{2}cos\left(\frac{\delta }{2}\right)\right)sin\left(\delta \right)\\ z & = & \frac{R}{2}sin\left(\frac{\delta }{2}\right) \end{array} \end{array}$$ (65)

Where *R* is the radius in x-y plane describing the width of the Mobius shape.

### NED to body frame block

This block is responsible for transforming the position control signals in n-frame to b-frame. The equations are:

$$\begin{array}{c} \begin{array}{ccc} \upsilon_{o}^{b} & = & R^{-1}\dot{P}\\ \omega_{nb}^{b} & = & T^{-1}\dot{\Theta } \end{array} \end{array}$$ (66)

Where *R* and *T* are shown in matrix notations Eqs (5) and (6).

### Inverse kinematics control model

This block is responsible for generating the reference angular speed in *rad*/*s* for each thruster corresponding to the desired AUV motion based on Eqs (9) and (10).

### Velocity and position controllers

**Fig 12:**
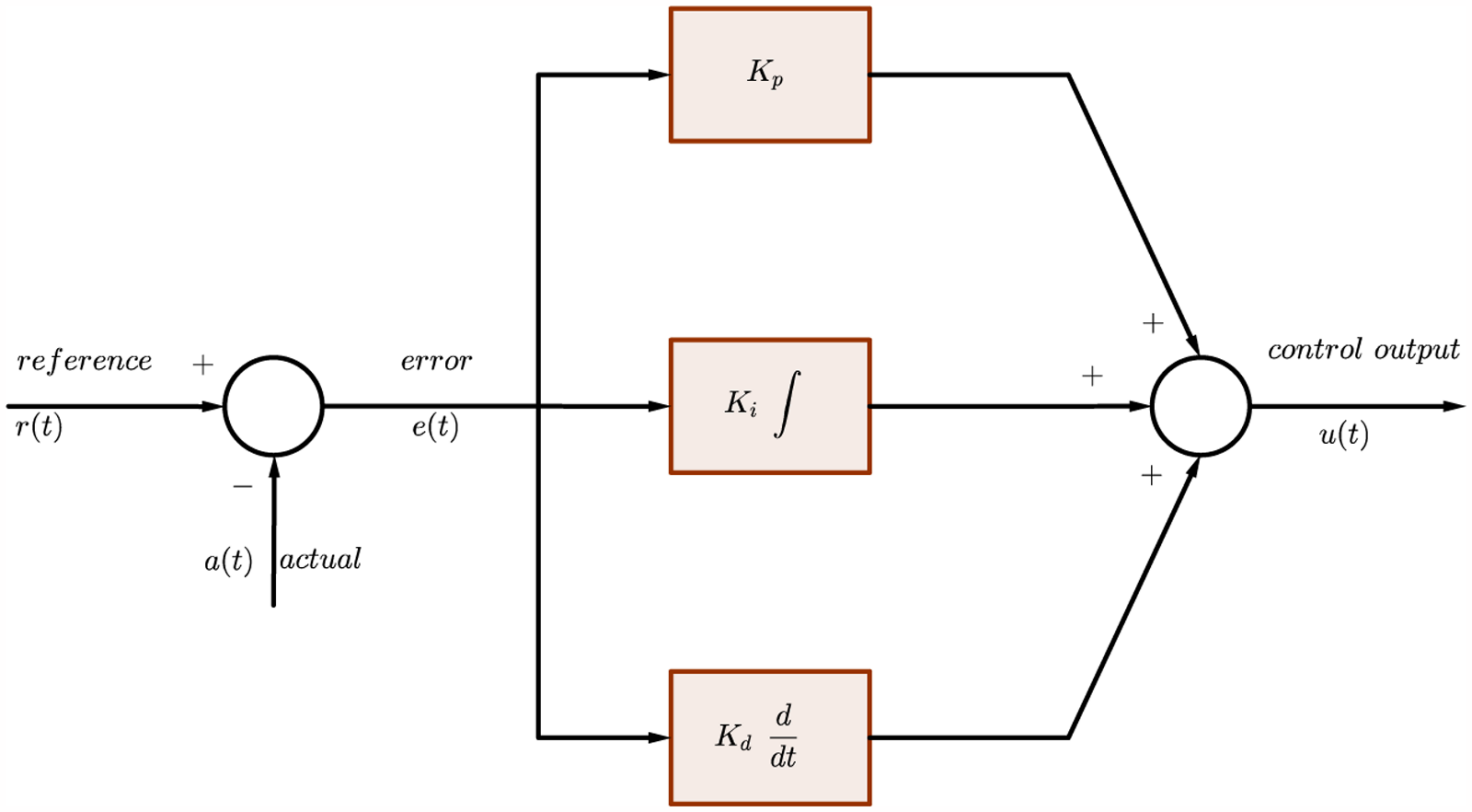
Continuous-time PID controller block diagram.

The PID controller used in this research is of the parallel form as shown in Fig 12.

The representation in continuous-time domain can by expressed as:

$$\begin{array}{c} u\left(t\right)=K_{p}\;e\left(t\right)+K_{i}\;\int_{0}^{t}\;e\left(\tau \right)d\tau +K_{d}\;\frac{de(t)}{dt} \end{array}$$ (67)

Regarding the discrete-time controller Forward Euler method is selected for the integration and derivation as they are shown in:

$$\begin{array}{c} \begin{array}{c} Discrete-time\;integration\;\left(Forward\;Euler\right)\Rightarrow \frac{Ts}{\left(z-1\right)}\\ Discrete-time\;derivation\;\left(Forward\;Euler\right)\Rightarrow \frac{N}{1+\frac{N.Ts}{\left(z-1\right)}} \end{array} \end{array}$$ (68)

The resultant discrete-time PID controller is represented by

$$\begin{array}{c} u\left(z\right)=K_{p}+K_{i}\;\frac{Ts}{\left(z-1\right)}+K_{d}\;\frac{N}{1+\frac{N\;Ts}{\left(z-1\right)}} \end{array}$$ (69)

Such that *K*_*p*_, *K*_*i*_, and *K*_*d*_ are the proportional, integral, and derivative gains, respectively. *u*(*z*) is the control output. *Ts* is the sampling time. *N* is a scaling factor.

**Fig 13:**
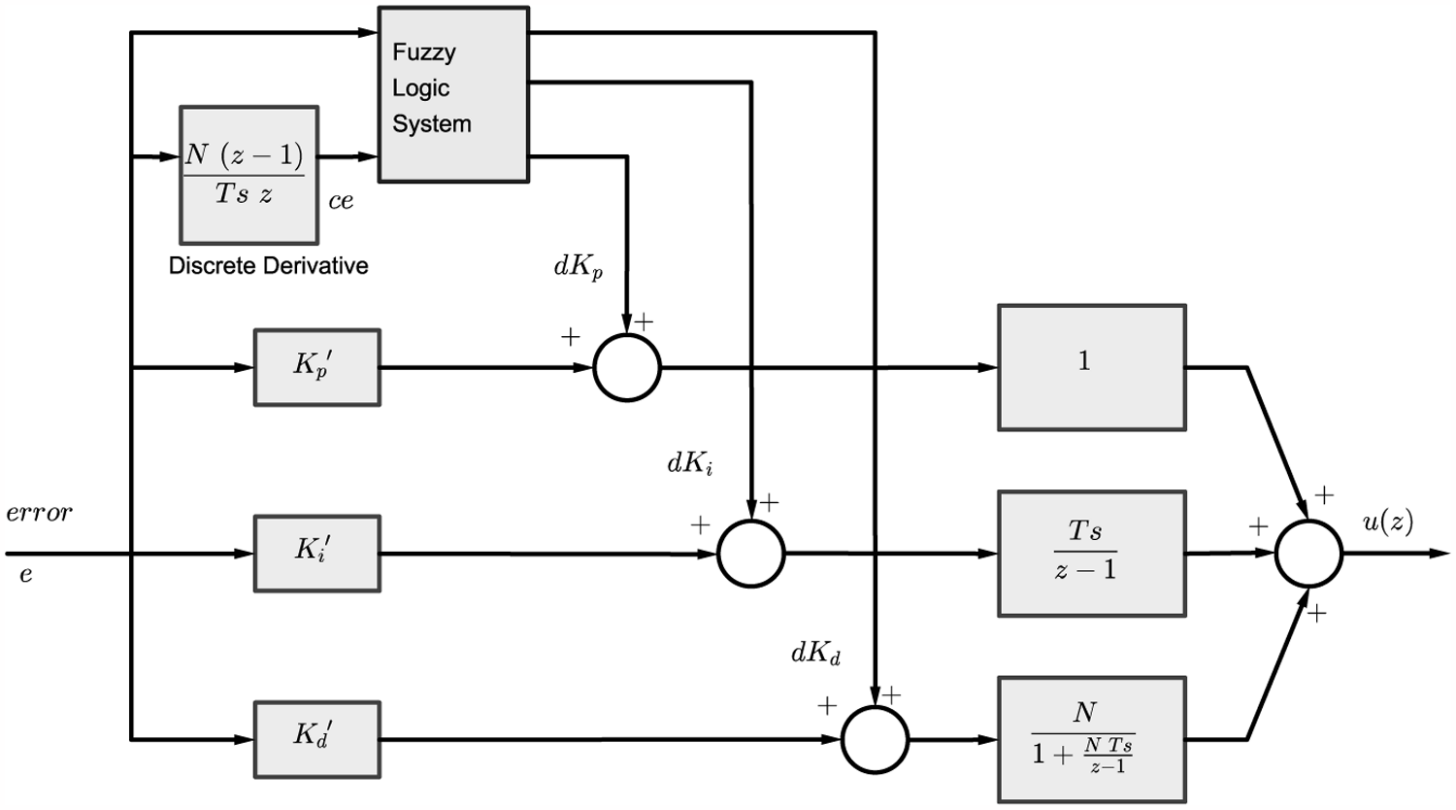
Discrete-time STFPID controller block diagram.

A Self-Tuned Fuzzy PID (STFPID) is designed as shown in Fig 13 such that the fuzzy inference system tunes the PID parameters intelligently based on the fuzzy rules of the expertise. Where the inputs to the fuzzy inference system are the error *e* and change in error *ce* and the outputs are *dK*_*p*_, *dK*_*i*_, and *dK*_*d*_.

The new STFPID tuning parameters can be expressed by:

$$\begin{array}{c} \begin{array}{ccc} K_{p}^{\prime } & = & K_{p}+dK_{p}\\ K_{i}^{\prime } & = & K_{i}+dK_{i}\\ K_{d}^{\prime } & = & K_{d}+dK_{d} \end{array} \end{array}$$ (70)

**Fig 14:**
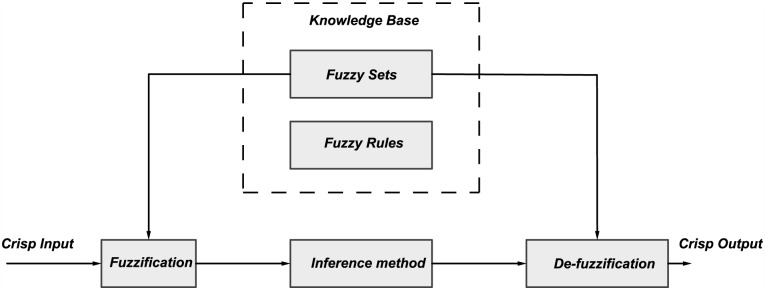
Fuzzy logic system.

The purpose of fuzzy logic is to formalize and implement a human being’s method of reasoning. It can therefore be classified as a field of artificial intelligence. The fuzzy rule base tool is the most common tool that is used in control applications. It is made of rules based on the human expertise. A number of rules have been defined based on the experiments and expertise to tune the PID parameters based on the knowledge of error and change in the error. These inputs are fuzzified as a first step. Then a reasoning is performed based on the rules defined, and degree of activation is calculated for each rule that depends on the classes the fuzzified inputs belong to. After that implication is performed. Aggregation is done to compute the final fuzzified output from the outputs of each rule. Finally, the output is de-fuzzified to obtain a crisp output that can be used to tune the PID parameters as illustrated in Fig 14.

As a result, the discrete-time PID represented by Eq (69) can be reformulated such that

$$\begin{array}{c} \begin{array}{ccc} u(z) & = & K_{p}^{\prime }+K_{i}^{\prime }\;\frac{Ts}{\left(z-1\right)}+K_{d}^{\prime }\;\frac{N}{1+\frac{N\;Ts}{\left(z-1\right)}}\\ & = & \left[K_{p}+dK_{p}\right]+\left[K_{i}+dK_{i}\right]\;\frac{Ts}{\left(z-1\right)}+\left[K_{d}+dK_{d}\right]\;\frac{N}{1+\frac{N\;Ts}{\left(z-1\right)}} \end{array} \end{array}$$ (71)

To generate the *dK*_*p*_, *dK*_*i*_, and *dK*_*d*_ a Fuzzy rules table has been proposed in Tables 3–5, respectively, based on the expertise. The fuzzy linguistic variables are Negative Big (NB), Negative Medium (NM), Negative Small (NS), Zero (ZO), Positive Small (PS), Positive Medium (PM) and Positive Big (PB).

**Table 3:** *dK*_*p*_ fuzzy rule table.

ce	NB	NM	NS	ZO	PS	PM	PB
e							
**NB**	ZO	ZO	NS	NS	PS	ZO	ZO
**NM**	NS	NS	NM	NM	NS	ZO	NS
**NS**	PS	ZO	NS	PS	ZO	ZO	NS
**ZO**	PM	PM	PS	ZO	NS	NM	NM
**PS**	PS	PS	ZO	NS	NM	NM	NM
**PM**	PS	ZO	NS	NS	NM	NM	NB
**PB**	ZO	ZO	NM	NM	NB	NB	NB

**Table 4:** *dK*_*i*_ fuzzy rule table.

ce	NB	NM	NS	ZO	PS	PM	PB
e							
**NB**	PB	PB	PB	PB	PB	PB	PB
**NM**	PB	PB	PB	PB	PB	PB	PB
**NS**	NB	NM	NM	PB	PB	PB	PS
**ZO**	NB	NM	NS	ZO	PS	PM	PB
**PS**	ZO	ZO	PS	PS	PM	PM	PB
**PM**	ZO	ZO	PS	PM	PM	PB	PB
**PB**	ZO	ZO	PS	PM	PM	PB	PB

**Table 5:** *dK*_*d*_ fuzzy rule table.

ce	NB	NM	NS	ZO	PS	PM	PB
e							
**NB**	PB	PS	NB	NB	NB	NM	PB
**NM**	PB	NM	NB	NB	NM	NM	ZO
**NS**	PM	PB	NM	NB	NS	PS	PS
**ZO**	ZO	NS	NS	ZO	NS	NS	ZO
**PS**	ZO	ZO	ZO	ZO	ZO	ZO	ZO
**PM**	PS	NB	PB	PB	NM	NS	NB
**PB**	PB	NS	NB	NB	NB	NM	PB

When the deviation |*e*| is large, in order to have fast-tracking performance, *k*_*p*_ should be greater. Taking a smaller value of *k*_*d*_ prevents instantaneous value of |*ec*| to be too large, at the same time a larger system response in order to avoid the overshoot, the integral action should be limited, the *k*_*i*_ value should normally be very small.

When the deviation |*e*| is of medium size, in order to ensure fast system response and have small overshoot, *k*_*p*_ should be reduced. Larger *k*_*d*_ increases the impact of system response, *k*_*i*_ should be appropriate.

When |*e*| is small, *k*_*p*_ and *k*_*i*_ should be bigger to ensure that the system has the ideal static performance. To avoid the vicinity of a shock at the system settings, *k*_*d*_ shall be chosen by the change of |*e*|.

**Fig 15:**
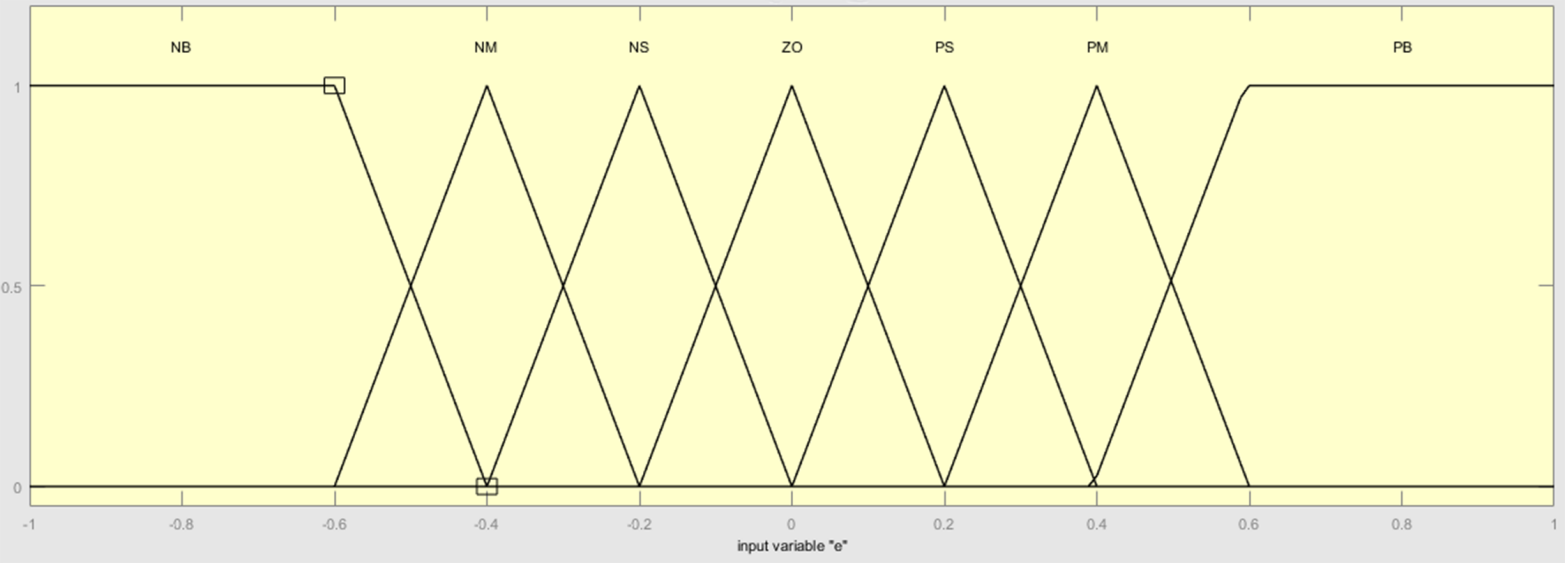
Inputs membership functions.

**Fig 16:**
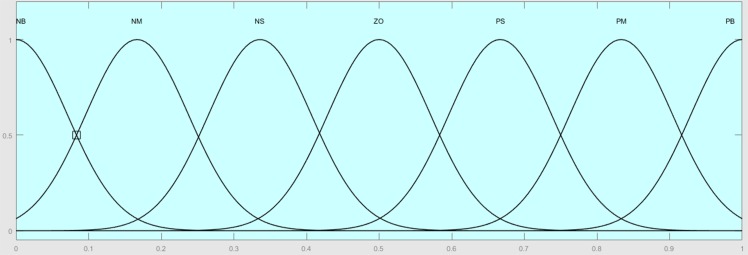
Output membership functions.

The membership functions for the inputs are *trapmf* and *trimf*, which represent trapezoidal and triangular membership functions, respectively, as shown in Fig 15. The output is *gaussmf*, which represents Gaussian membership function as shown in Fig 16.

**Fig 17:**
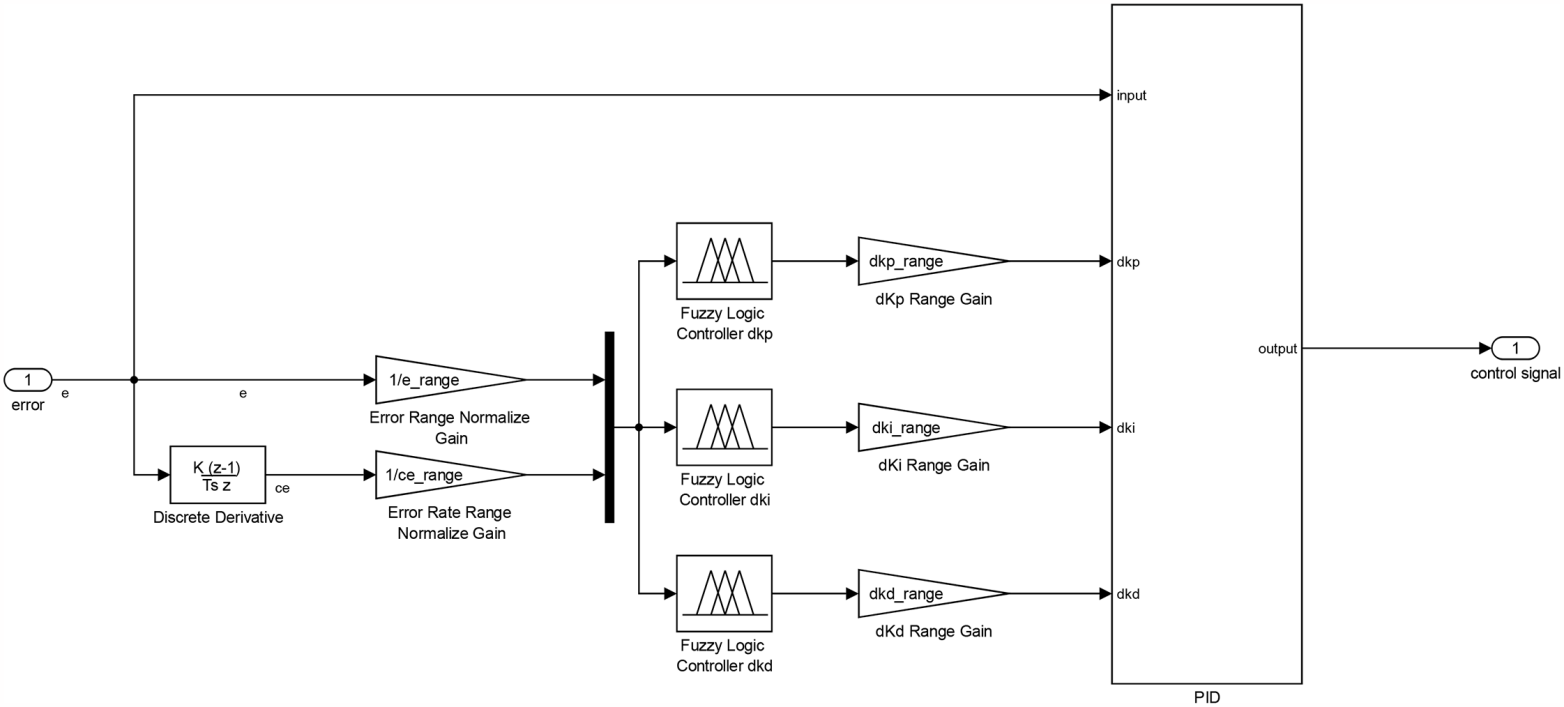
STFPID controller schematic.

**Fig 18:**
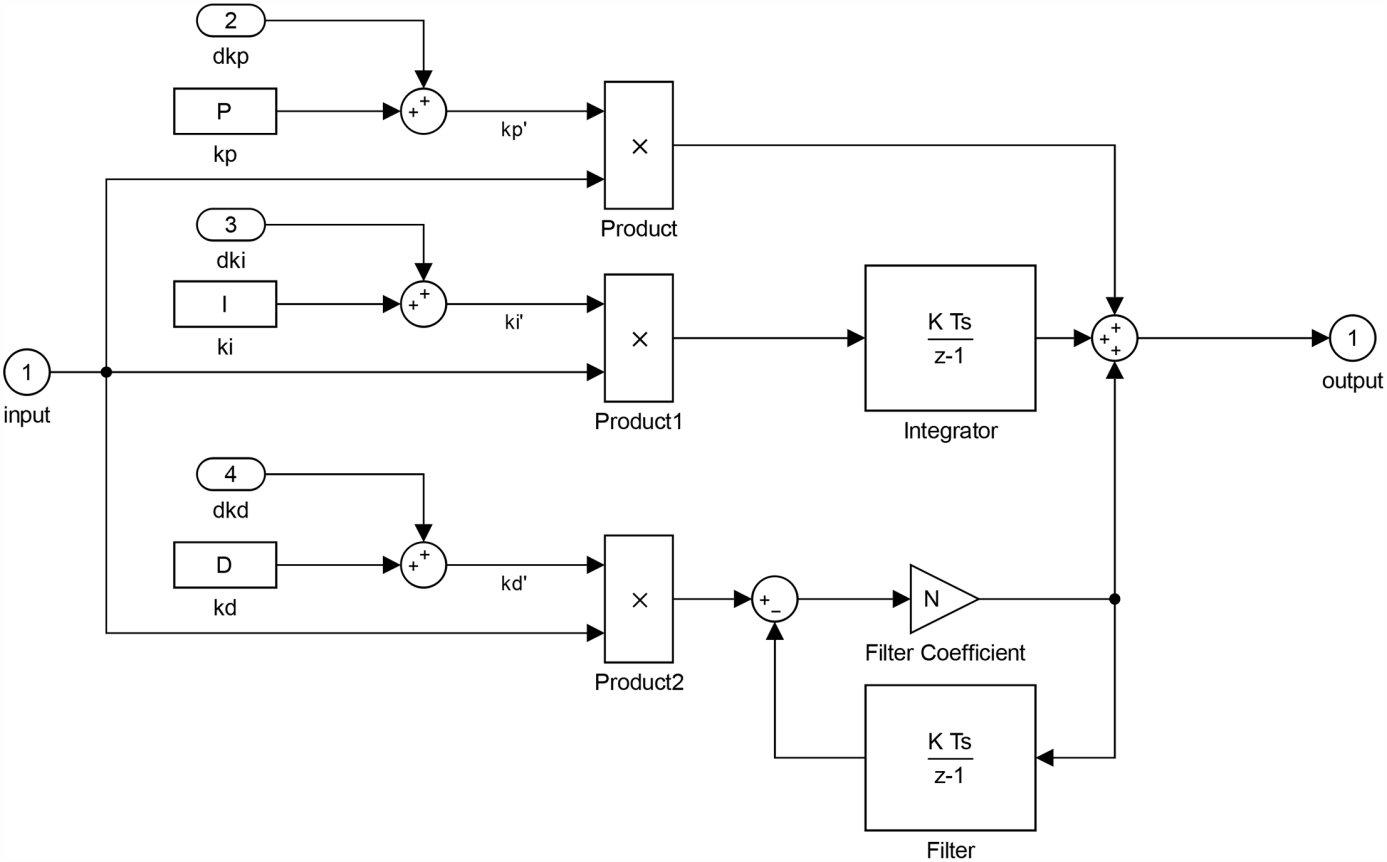
PID.

The final schematic for the STFPID including the fuzzy inference system is as shown in Figs 17 and 18. The inputs and outputs of the fuzzy inference system are normalized by ranging factors that describe the ranges of inputs and outputs.

### AUV system

**Fig 19:**
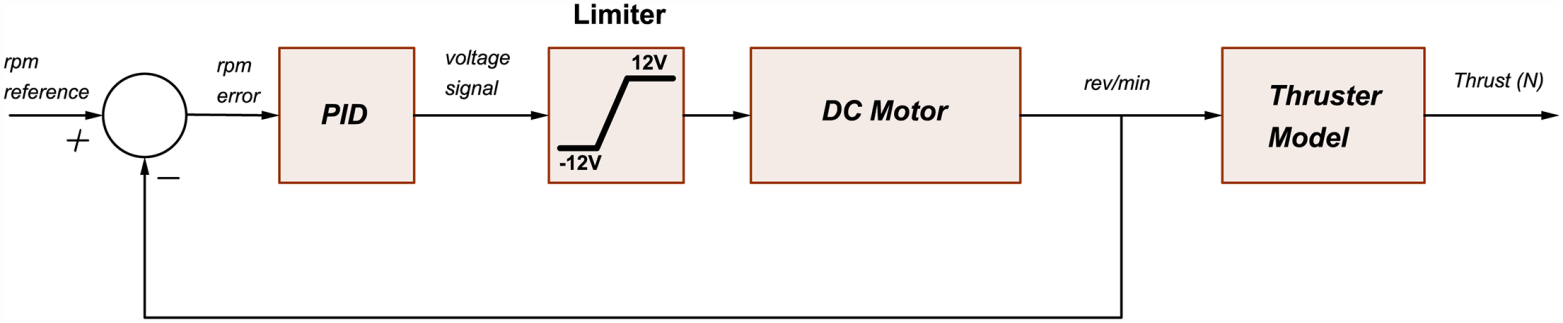
Thruster control loop.

This block is used only for simulation and consists of the model of eight thrusters actuator as listed in Eqs (50)–(53), such that a closed loop control using conventional PID are implemented for each thruster as shown in Fig 19.

The thruster model equations are that listed in Eqs (54) and (55). The block also consists of the thrusters-vessel forward kinematics listed in Eqs (56)–(61). These calculate the resultant forces and moments on the AUV resulting from each thruster’s propeller rotation. Finally, it also contains the dynamic model of the AUV formulated in Eq (20), but is reformulated as shown in Eq (72) such that the inputs are the forces acting on the AUV body frame and the outputs are the vessel velocity states, as shown in the hydrodynamic environment model shown in Eqs (27), (29) and (30).

$$\begin{array}{c} \begin{array}{ccc} \dot{u} & = & \frac{X}{m}+vr-wq\\ \dot{v} & = & \frac{Y}{m}+wp-ur\\ \dot{w} & = & \frac{Z}{m}+uq-vp\\ \dot{p} & = & \frac{1}{I_{x}}\;[K-(I_{z}-I_{y})qr]\\ \dot{q} & = & \frac{1}{I_{y}}\;[M-(I_{x}-I_{z})rp]\\ \dot{r} & = & \frac{1}{I_{z}}\;[N-(I_{y}-I_{x})pq] \end{array} \end{array}$$ (72)

## Results

The simulation of this research is implemented and validated using Mathworks Simulink. The system specifications and parameters that have been chosen are as demonstrated in the Table 6.

**Table 6:** System specifications and values used for the simulation.

Parameter	Value
Sampling Time (dt)	0.001 (s) or 1 (ms)
AUV length	0.4 (m)
AUV width	0.3 (m)
AUV height	0.3 (m)
Origin-horizontal thruster distance (*l*_*h*_)	0.17 (m)
Origin-vertical thruster distance (*l*_*v*_)	0.17 (m)
Propeller Pitch (*P*_*prop*_)	0.1 (m/rev)
Control Factor (λ)	0.04 (unit-less)

The parameters of both velocity and position controllers have been selected as demonstrated in Tables 7 and 8, respectively.

**Table 7:** Velocity controller parameters.

Parameter	u Control	v Control	w Control	p Control	q Control	r Control
*K*_*p*_	1	1.5	1	1	1	1
*K*_*i*_	1	1.5	1	1	1	1
*K*_*d*_	0.1	0	0	0	0	0
*e range*	2	2	2	2	2	2
*ce range*	500	500	500	200	200	200
*dk*_*p*_ *range*	2	2	2	2	2	2
*dk*_*i*_ *range*	2	2	2	2	2	2
*dk*_*d*_ *range*	0	0	0	0	0	0

**Table 8:** Position controller parameters.

Parameter	x Control	y Control	z Control	*ϕ* Control	*θ* Control	*ψ* Control
*K*_*p*_	5	5	5	10	10	10
*K*_*i*_	0	0	0	0	0	0
*K*_*d*_	10	10	10	10	10	10
*e range*	4	1	1	0.7	0.7	0.7
*ce range*	400	400	400	0.05	0.05	0.05
*dk*_*p*_ *range*	20	20	20	10	10	10
*dk*_*i*_ *range*	0	0	0	0	0	0
*dk*_*d*_ *range*	0	0	0	0	0	0

**Fig 20:**
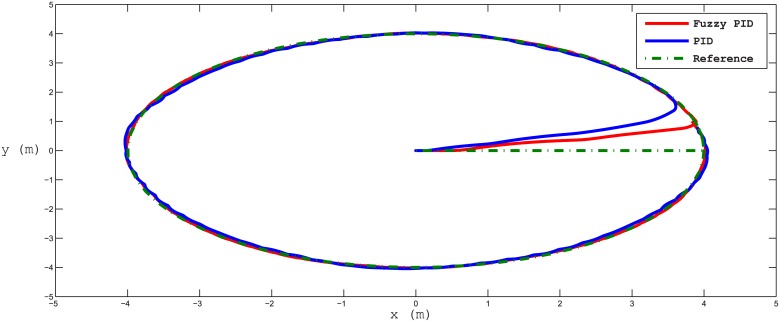
x-y plan circle trajectory scenario (without disturbances). The red trajectory is the output from the fuzzy PID controller, the blue trajectory is the output from the PID controller, and the green-dotted line is the reference trajectory.

**Fig 21:**
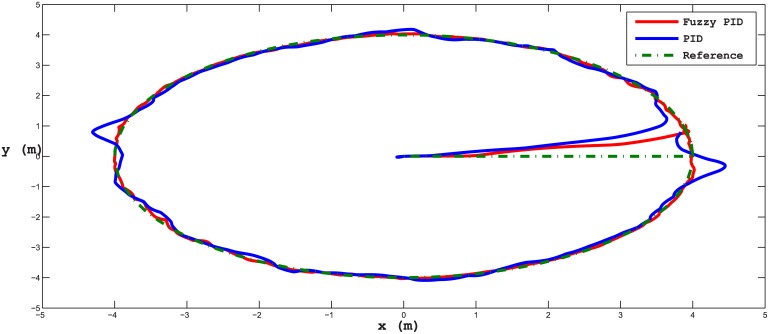
x-y plan circle trajectory scenario (with disturbances). The red line is the output from the fuzzy PID controller, the blue line is the output from the PID controller, and the green-dotted line is the reference.

**Fig 22:**
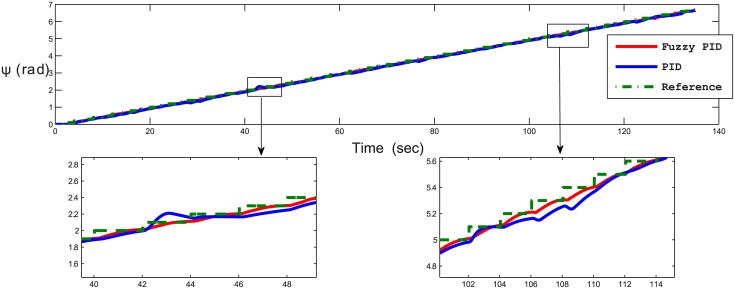
Time response of yaw orientation of the x-y plan circle trajectory scenario (without disturbances). The red line is the fuzzy PID yaw angle output, the blue line is the PID yaw angle output, and the green-dotted line is the reference yaw angle.

**Fig 23:**
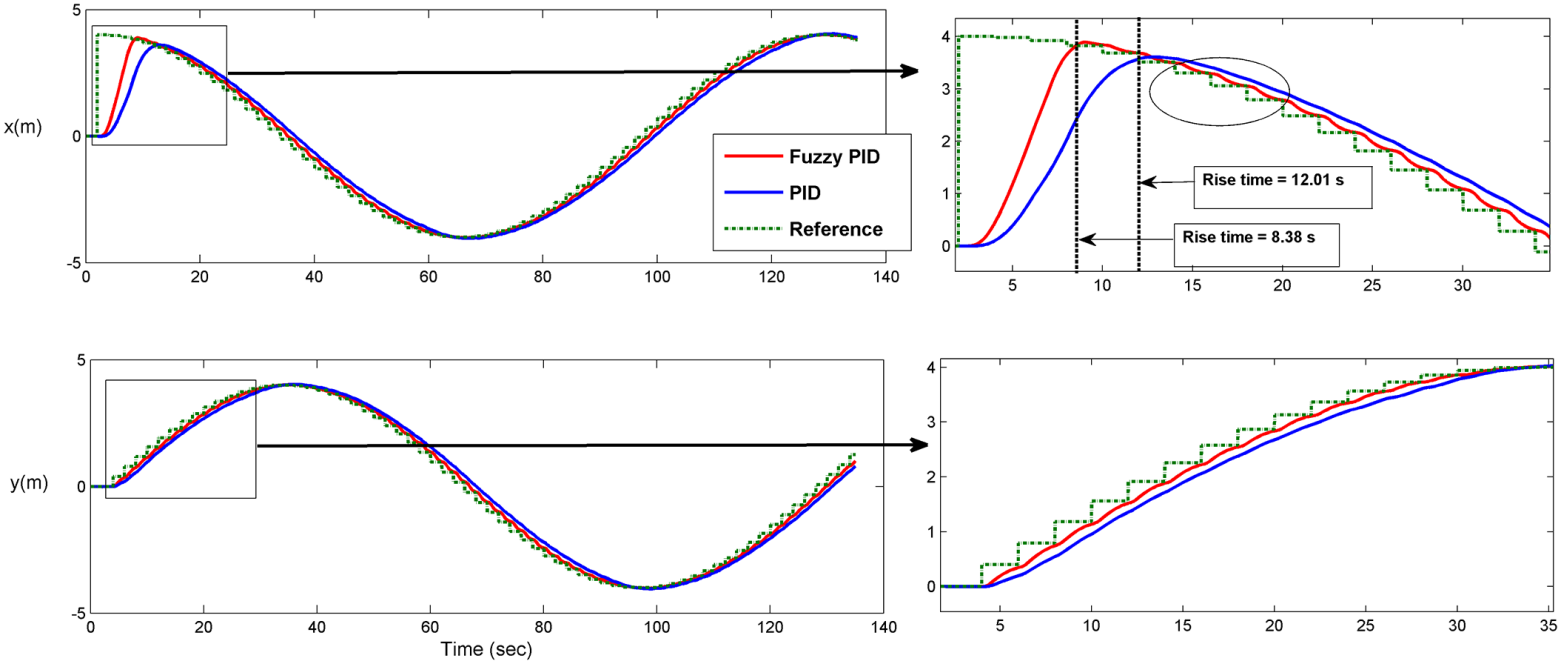
Time response of x-y plan circle trajectory scenario (without disturbances). The red line is the output from the fuzzy PID controller, the blue line is the output from the PID controller, and the green-dotted line is the reference.

**Fig 24:**
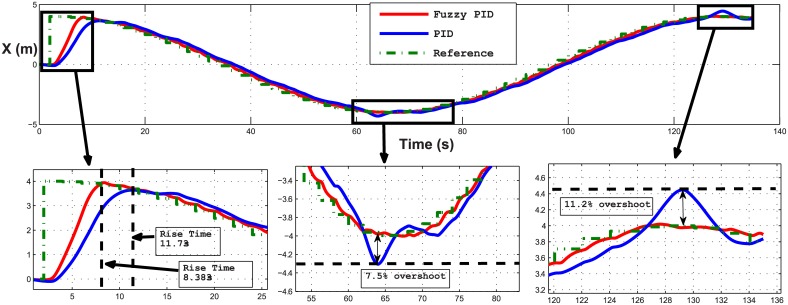
Time response of x position of the circle trajectory scenario (with disturbances). The red line is the output from the fuzzy PID controller, the blue line is the output from the PID controller, and the green-dotted line is the reference.

Several test scenarios have been executed to validate the control model using different trajectories with and without ocean disturbances. The results shown in the figures below are for STFPID performance relative to conventional PID. In the first test scenario a circle trajectory in x-y has been generated with and without disturbances, the results are shown in Figs 20 and 21. Fig 22 shows the performance of following the reference of the yaw orientation. Figs 23 and 24 demonstrate the time response of the xy positions for the system without and with disturbances, respectively. From a time response perspective, Figs 20 and 23 show that STFPID response is faster to achieve the reference waypoints such that the rising times are *T*_*s*_ = 12.01*s* and *T*_*s*_ = 8.38*s* for PID and STFPID, respectively. Figs 21 and 24 show that the overshoots are ranges between 7.5% and 11.2% in the case of PID, which means that the STFPID has much better disturbance attenuation capability. The STFPID response time is better, at *T*_*s*_ = 8.383*s* as compared to *T*_*s*_ = 11.73*s* for PID. The data sets of the test scenario for circle trajectory in xy-plane with injecting disturbances can be found in S1 File.

**Fig 25:**
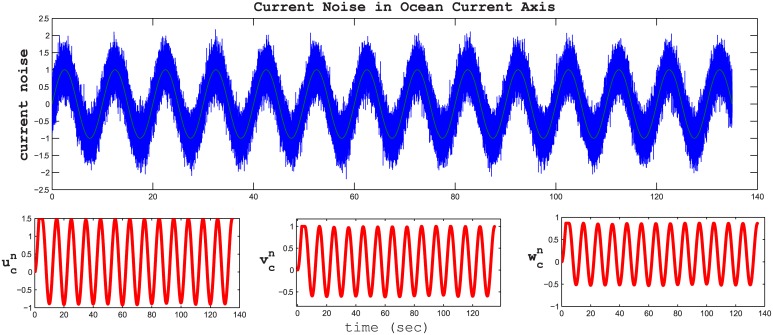
Disturbance effect in n-frame coordinates. The sinusoidal blue wave is the noise wave in the current disturbance reference frame. The red waves in the three below figures are the current disturbances in the inertial n-frame axes.

**Fig 26:**
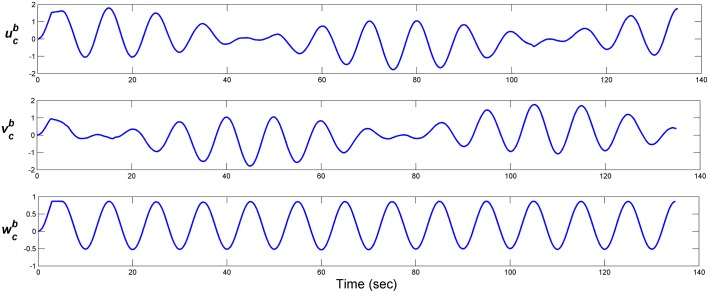
Disturbance effect in b-frame coordinates. The disturbances in the body reference frame.

The noise wave form is a sinusoidal wave with additive white Gaussian noise as shown in Fig 25 with a frequency of 0.1 Hz and a signal to noise ratio (SNR) 10 dB. The resultant ocean current in n-frame and b-frame are as shown in Figs 25 and 26, respectively.

**Fig 27:**
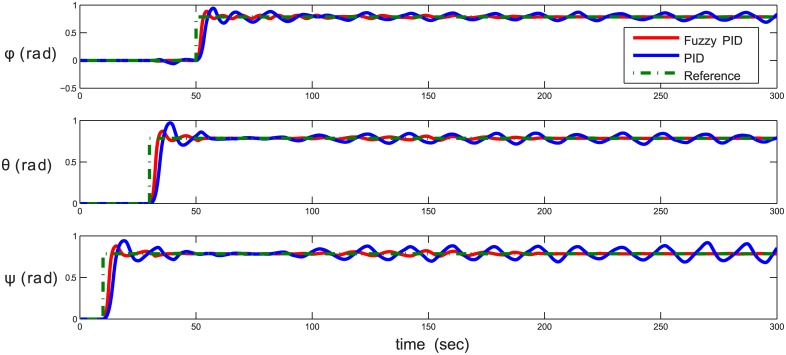
Attitude control (yaw-pitch-roll sequence). The red line is the output from the fuzzy PID controller, the blue line is the output from the PID controller, and the green-dotted line is the reference.

The second test scenario is an attitude control for yaw, pitch, and roll successively without injecting disturbances as demonstrated in Fig 27. For attitude and orientation control, the STFPID also proves a significant improvement in achieving the reference attitude with almost no oscillations and very small overshoots compared to conventional PID. It also shows stability over time, while in case of PID, the system starts to oscillate due to system non-linearity.

**Fig 28:**
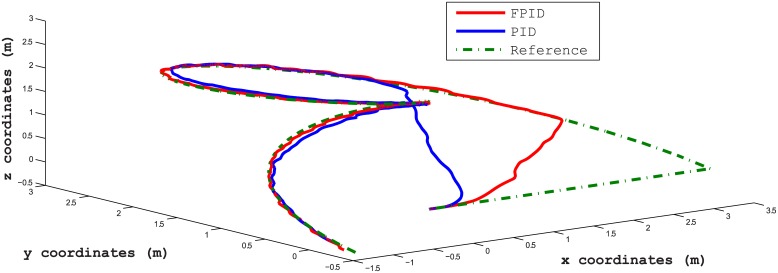
Trajectory following of a Mobius shape trajectory in 3D environment. The red line is the output from the fuzzy PID controller, the blue line is the output from the PID controller, and the green-dotted line is the reference.

**Fig 29:**
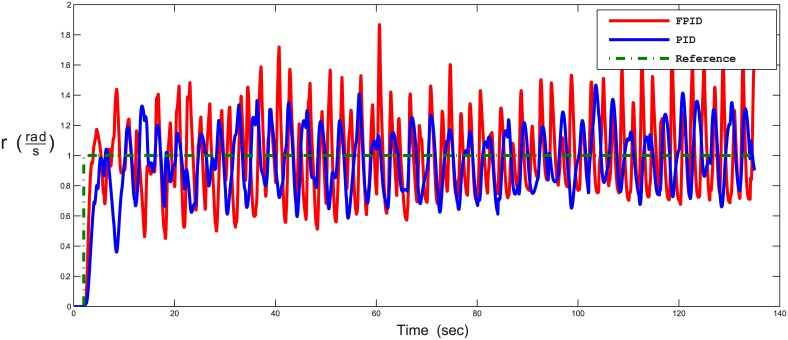
Time response of the angular velocity about b-frame z-axis in following Mobius trajectory. The red line is the output from the fuzzy PID controller, the blue line is the output from the PID controller, and the green-dotted line is the reference.

**Fig 30:**
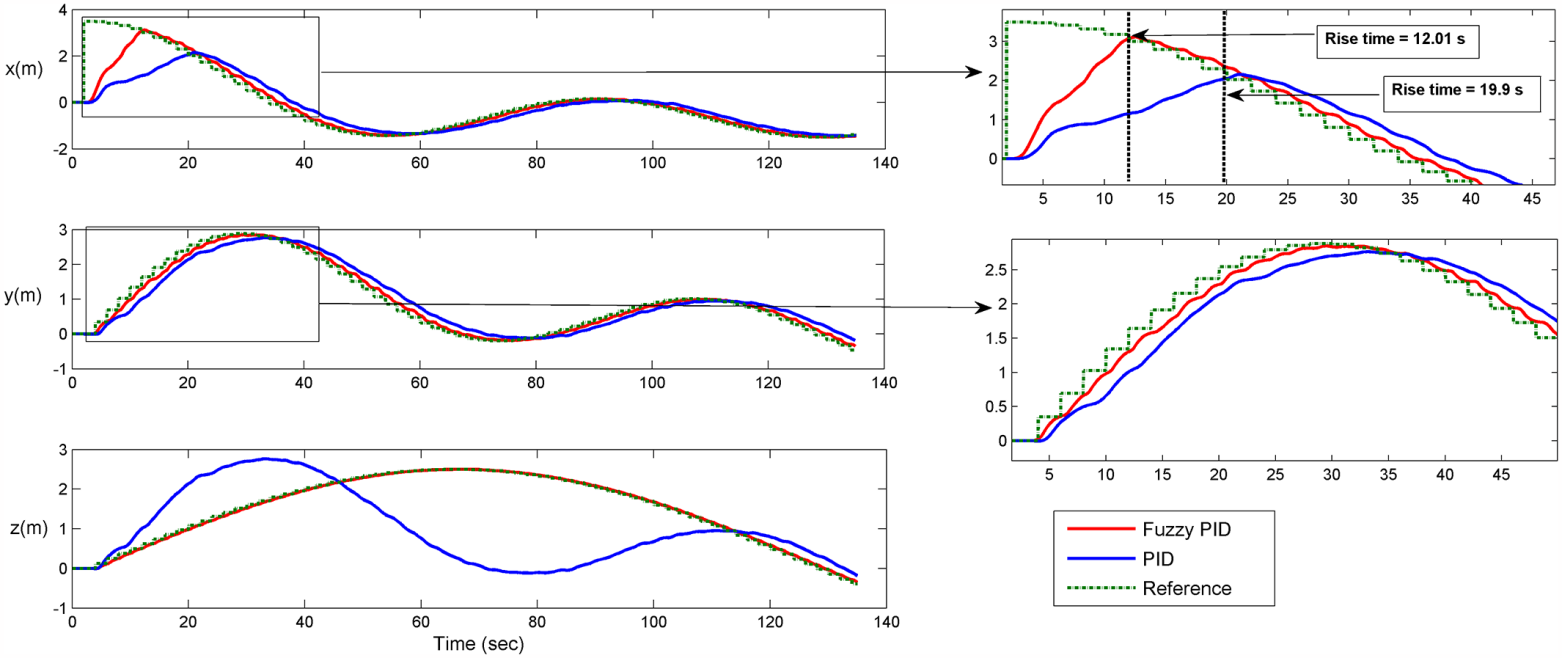
Time response of the xyz positions of Mobius trajectory. The red line is the output from the fuzzy PID controller, the blue line is the output from the PID controller, and the green-dotted line is the reference.

The third test scenario is for validating separate control of speed and position at the same time. In this test, the AUV is controlled such that it follows a trajectory with a Mobius shape in the 3D plan, along with having a constant angular rate around the AUV z-axis [*p* angular velocity]. There were no disturbances in this scenario. The results for following the way-points in xyz plan and time-response of angular velocity are shown in Figs 28 and 29, respectively. Fig 30 shows the time response of the xyz positions where the STFPID reached the reference way-points at rising time equal to 12 s while the PID reached the reference at more than 20 s. The data sets can be found in S2 File.

The control architecture here proves that it is capable of isolating position and velocity control of any DOF. For example, one can control the position for x, y, and z DOFs, and at the same time control the angular velocity of the rotation about b-frame z-axis [*z*_*b*_] as presented in Figs 28 and 29. As Fig 29 demonstrates, the oscillations frequency in the angular velocity is very high and that’s because the position controller for x, and y DOFs are controlling the horizontal thrusters, which affects the yaw rate rotation. At the same time, the angular velocity control of [*r*] changes the speeds of the horizontal thrusters as listed in Eq (9). So both controllers are pushing against each others, but the angular velocity is oscillating around the reference velocity and not deviating to instability. In case of using the STFPID the oscillations amplitude is higher than that of conventional PID but its response time is faster.

## Conclusion

The designed STFPID controller coupled with the inverse kinematic control model studied in this research shows a significant improvement in the time-response performance in controlling a fully-actuated AUV with fast response and minimum error compared to conventional PID. STFPID also shows better performance, even when ocean current disturbances are injected to the AUV system with almost very small overshoots compared to conventional PID that had a very large overshoot and slow response time. Furthermore, the control architecture presented in this work shows that the double control loops make the system capable of controlling both velocity and position independently as desired by the user or the references.

## Supporting information

S1 File Data sets and script. This file contains the data sets and script to visualize the scenario XY Circle with disturbances.
(RAR)

S2 File Data sets and script. This file contains the data sets and script to visualize the scenario XYZ Mobius trajectory.
(RAR)
